# Low-Temperature Exsolution of Rh from Mixed ZnFeRh Oxides toward Stable and Selective Catalysts in Liquid-Phase Hydroformylation

**DOI:** 10.1021/jacs.4c14839

**Published:** 2025-02-10

**Authors:** Daniel Delgado, Gregor Koch, Shan Jiang, Jinhu Dong, Jutta Kröhnert, Franz-Philipp Schmidt, Thomas Lunkenbein, Carmen Galdeano Ruano, José Gaona-Miguélez, Diego Troya, Pascual Oña-Burgos, Annette Trunschke

**Affiliations:** † Department of Inorganic Chemistry, Fritz-Haber-Institut der Max-Planck-Gesellschaft, 14195 Berlin, Germany; ‡ Instituto de Tecnología Química, Universitat Politècnica de València-CSIC, 46022 Valencia, Spain; § Department of Chemistry, Virginia Polytechnic Institute and State University, Blacksburg, Virginia 24061, United States

## Abstract

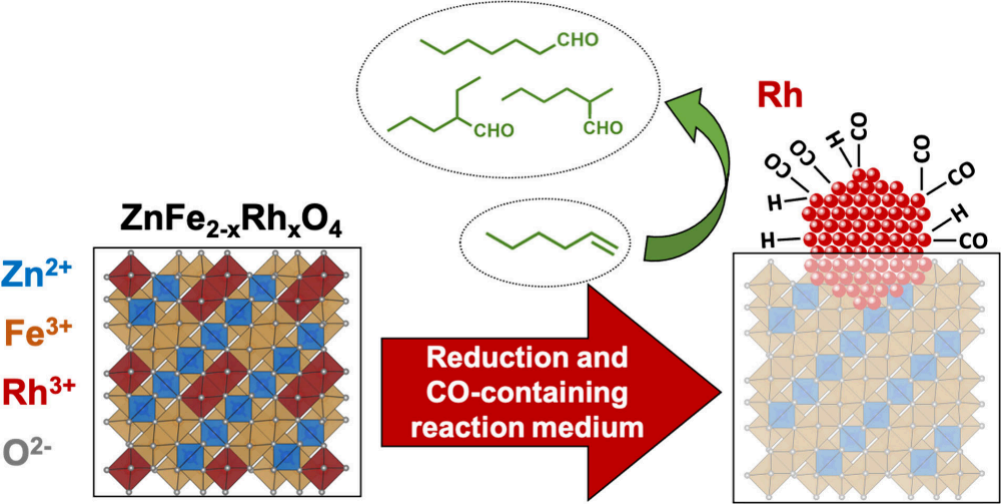

The exsolution of metal nanoparticles offers a promising strategy to enhance catalyst stability and fine-tune metal–support interactions. Expanding the use of exsolved nanoparticles in heterogeneous catalysis requires the development of low-temperature (*T* < 400 °C) exsolution processes. In this study, we report the synthesis of phase-pure ZnFe_2–*x*_Rh_*x*_O_4_ metal oxide precursors with a spinel-type crystal structure. The isomorphic substitution of Fe^3+^ in the host lattice by Rh^3+^ was confirmed by X-ray diffraction and Raman spectroscopy combined with DFT calculations. The hydrothermal synthesis method of the oxide precursors was specifically chosen so that very small oxide particles of 10–20 nm were obtained, which enabled the exsolution of Rh nanoparticles with a particle size of about 1 to 2 nm at temperatures below 200 °C in a hydrogen-containing atmosphere. Compared to a Rh catalyst prepared by conventional wet impregnation of ZnFe_2_O_4_, the catalysts obtained by low-temperature exsolution show superior properties in terms of selectivity toward aldehydes in the hydroformylation of 1-hexene in the liquid phase. In addition, there is no Rh loss due to leaching, which is the main challenge for heterogeneous Rh catalysts used in liquid phase reactions. The exceptionally strong metal–support interaction imparts unique nanostructures and electronic properties to the exsolved metal nanoparticles, as revealed by electron energy loss spectroscopy (EELS) and diffuse reflectance infrared Fourier transform (DRIFT) spectroscopy. The specific adsorption sites on the exsolved Rh particles lead to stronger metal–hydride and weaker metal–carbonyl bonds on the surface, steering the reaction pathway toward hydroformylation rather than olefin isomerization.

## Introduction

Solid catalysts are complex materials with dynamic behavior when exposed to the chemical potential of the feed under activation and reaction conditions.^1−7^ The design of functional interfaces for a specific chemical transformation is therefore one of the key challenges in the synthesis of active and selective catalysts in heterogeneous catalysis. In this context, the exsolution of metallic species from well-defined metal oxide precursors, i.e., precursors with defined composition and crystal structure, stands as a synthetic strategy that ensures a better control of the chemical nature of the catalyst precursor, i.e., prior to activation and subsequent reaction.^8^ This technique leads to unique functional properties derived from the strong metal–support interaction between the exsolved particles and the metal oxide host structure.^9^

Among the benefits of exsolved metal nanoparticles in terms of catalytic applications, the improved catalyst stability, due to the socketed nature of the metal nanoparticles, makes these materials suitable for reactions in which particle sintering^10,11^ and deactivation due to coke deposition^12^ stand as important issues. However, there are some drawbacks that prevent a wider application of exsolved nanoparticles in heterogeneous catalysis, including the low surface areas of the metal oxide precursors and/or high temperature reduction treatments applied to exsolve the metallic particles, which also generate low metallic surface areas.^13,14^ To increase both the metal and support surface areas, low temperature reduction treatments in combination with small particle size metal oxide precursors could, in principle, lead to small metal particles anchored to high surface area metal oxide supports.

In this work, we have developed a series of ZnFe_2–*x*_Rh_*x*_O_4_ metal oxides with a spinel structure prepared by hydrothermal synthesis able to exsolve Rh at low temperatures, up to 200 °C. Rhodium is of particular interest as a catalytically active metal in hydrogenation and hydroformylation reactions that are performed at temperatures below 200 °C. Hydroformylation of alkenes with syngas (CO + H_2_) is one of the most relevant sources of aldehydes in the chemical industry (ca. 10 MT/year) and is still carried out using Rh-phosphine/phosphite complexes as homogeneous catalysts.^15−18^ In the last decades, a vast number of reports have been focused on the development of Rh-containing solid catalysts that can emulate the catalytic properties of their homogeneous counterparts.^16,17^ Among the problems of solid catalysts in hydroformylation, the leaching of Rh species, especially in liquid-phase reactions, is probably the most important issue, together with the lower catalytic activity shown compared to Rh-phosphine/phosphite homogeneous catalysts.^19^ Synthetic strategies toward the preparation of heterogeneous catalysts based on Rh for hydroformylation include supported Rh complexes,^20^ supported Rh nanoparticles^21^ and single atoms,^22−24^ Rh-metal oxide dual sites,^25^ or Rh phosphide-based catalysts.^26−28^ Interestingly, to date there is no example of the application of metal exsolution as synthetic strategy for the synthesis of active catalysts in liquid-phase reactions in thermal catalysis, being most of the reports devoted to high temperature gas-phase processes, for which exsolution has demonstrated to be a method to improve catalytic performance and avoid particle sintering.^9^

The aim of the present work was to investigate whether the exsolution strategy could effectively prevent the loss of the active metal to the liquid phase during catalytic reactions at the solid–liquid interface in thermal catalysis. Furthermore, it can be a priori assumed that the improved metal–support interaction driven by the exsolution of metal nanoparticles could also improve the catalyst performance in liquid-phase processes. Therefore, the influence of the preparation method on the catalysis was investigated. Indeed, the comparison of spinel-supported Rh nanoparticles obtained by exsolution with ZnFe_2_O_4_-supported Rh catalysts prepared by conventional wet impregnation reveals an enhanced interaction of exsolved Rh nanoparticles with the support, resulting in resistance to leaching, stronger Rh–H surface bonds, and more labile Rh-CO surface species, thus leading to stable operation and higher selectivity toward aldehydes in the hydroformylation of 1-hexene. These unique catalytic properties are attributed to the very low exsolution temperature of 200 °C, which to our knowledge is the lowest reported in the literature up to date, for such a material system.

## Methods

### Catalyst Synthesis

A series of ZnFe_2–*x*_Rh_*x*_O_4_ (x= 0–0.1) materials has been synthesized by a hydrothermal protocol based on the decomposition of oxalates,^29−31^ using zinc sulfate heptahydrate (Carl Roth, 99.5%, Lot: 139102089), iron(III) chloride anhydrous (Sigma-Aldrich, Lot: S8066245), Rh chloride hydrate (Sigma-Aldrich, 38%, Lot: MKCR2868), oxalic acid dihydrate (Sigma-Aldrich, 99%, Lot: MKCM3794), sodium hydroxide (Sigma-Aldrich, 99%) and tetrabutylammonium hydroxide (AppliChem, 20% w/w in H_2_O, Lot: 0002053684). Aqueous solutions (132.6 mL of deionized water) containing stoichiometric amounts of the corresponding metal salts and oxalic acid (Zn^2+^/(Fe^3+^+Rh^3+^)/oxalic acid, 11.75/23.50/35.25 mmol) and a total metal concentration of 0.27 mol L^–1^ were basified with 10 M NaOH (pH = 10.3). Then, 23 mL of tetrabutylammonium hydroxide (20% w/w in H_2_O) was added and the obtained suspensions were introduced into an analytic autoclave HPM-PT-04 (400 mL) made of Hastelloy C22 (Premex Reactor GmbH) and treated at 140 °C for 12 h under autogenous pressure (heating rate: 3 °C·min^–1^, cooling rate: 1 °C·min^–1^, stirring rate: 100 rpm). The resulting suspensions were centrifuged to recover a solid fraction that was washed and centrifuged 6 consecutive times with ca. 240 mL of H_2_O. The washed solid fractions were dried at 80 °C for 20 h, and subsequently heat-treated in air at 500 °C for 2h in a muffle furnace (heating rate 3 °C·min^–1^). The calcined oxides are referred to as fresh metal oxides in the text. The samples were named as Rh-*y*, with *y* being the Rh/Zn+Fe+Rh atomic ratio determined by ICP OES (Table 1). Samples Rh-0, Rh-0.6, Rh-1.5 and Rh-3.0 correspond to the internal sample IDs S34398, S34366, S34367, and S34371, respectively, in an internal database, which are important to clearly identify reproductions of the synthesis and to be able to assign the experiments performed to the corresponding batch.

**Table 1:** Physicochemical Features of Phase-Pure^a^ ZnFe_2–*x*_Rh_*x*_O_4_ spinels

	Rh/(Zn+Fe+Rh)·100 (atom %)					
sample	nominal	experimental^b^	cell parameter (Å)^d^	*S*_BET_ (m^2^·g^–1^)^e^	average crystallite size (nm)^f^	total H_2_ uptake up to 900 °C (mmol_H2_·g^–1^)^g^
Rh-0	0	0	8.4430 (2)	49	11.95 (7)	12.43
Rh-0.6	0.8	0.6	8.4433 (2)	55	11.861 (4)	12.73
Rh-1.5	1.6	1.5	8.4444 (2)	62	12.17 (1)	12.30
Rh-3.0	3.3	3.0	8.4446 (2)	85	12.184 (3)	12.88
Rh/ZFO	0.8^c^	0.7	8.4430 (2)	43^h^	n.d.	12.28

a According to XRD. No secondary phases detectable in the patterns.

b Calculated from ICP OES of the fresh metal oxides.

c Calculated from 1 wt % Rh/ZnFe_2_O_4_.

d Estimated by Rietveld refinement using FullProf Suite.

e BET surface area calculated from N_2_-physisorption isotherms of the fresh metal oxides.

f Calculated by Williamson–Hall method.

g From TPR-H_2_ experiments.

h Determined from N_2_-adsorption isotherm of the ZnFe_2_O_4_ support.

ZnFe_2_O_4_-supported 1 wt % Rh catalyst (Rh/ZFO, internal sample ID S38188) was prepared by wet impregnation by adding the corresponding amounts of a Rh-containing solution (10% (w/w) HNO_3_ solution (5 wt % HNO_3_) diluted in water (1:107, on a weight basis)) to hydrothermally synthesized ZnFe_2_O_4_ (see above). The mixture was rotary evaporated at 60 °C for 1 h, and the resulting powder was kept at 80 °C for 72 h in a drying cabinet. Finally, the sample was treated at 500 °C (heating rate: 3 °C·min^–1^) for 2 h under air flow (100 mL·min^–1^) in a tubular furnace.

### Characterization Methods

X-ray diffraction patterns were collected in Bragg–Brentano geometry on a Bruker AXS D8 Advance II θ/θ diffractometer equipped with a Ni filter, and using Cu K_α1+2_ radiation.

Chemical analysis was carried out by means of Inductively Coupled Plasma Optical Emission Spectrometry (ICP OES). An Optima 8300 from PerkinElmer with Zyklon nebulizer was used in axial mode. The samples were dissolved in a multiwave Pro autoclave from Anton Paar, equipped with a Teflon liner, at 200 °C and 60 bar. Reagents in supra pure quality and water from an ELGA pure water system (VEOLIA) (conductivity 0.05 μS·cm^–1^) were used.

N_2_ adsorption experiments were carried out in an Autosorb-6B analyzer (Quantachrome). The metal oxides were outgassed at 200 °C for 1 h prior to the adsorption of N_2_ at −196 °C. The specific surface area *S*_*BET*_ was calculated according to the multipoint Brunauer–Emmett–Teller method (BET) in the *p/p*_0_ = 0.05–0.15 pressure range assuming a N_2_ cross sectional area of 16.2 Å^2^.

*Ex situ* Raman spectra were recorded with an WITec alpha300 R confocal Raman microscope (magnification: 50 × , N.D. 0.55) equipped with a 600 g·mm^–1^ grating and an excitation wavelength of 488 nm with a laser power of ∼1.5 mW at sample position. An integration time of 1 s and 100 accumulations were used to acquire the spectra. The Raman signals were calibrated with respect to the reference signal of a silicon wafer at a Raman shift of 520 cm^–1^.

*In situ* Raman experiments were carried out on a TriVista Raman Microscope system TR557 (S&I GmbH) equipped with a 750 mm monochromator (Princeton Instruments). A 488 nm laser source was introduced through an objective (50 × , N.D. 0.5) to the sample with 1 mW. Spectra were taken with 1000 s integration time and one accumulation. *In situ* measurements were conducted in a Linkam CCR1000 reaction cell (Linkam Scientific Instruments LTD). Fifteen mg sample was heated up with a heating rate of 1 °C·min^–1^ to 200 °C for 2 h in 5% H_2_/N_2_ (30 mL·min^–1^). The peak intensity ratios were read from the background-subtracted Raman spectra.

Scanning electron microscopy (SEM) images were collected using a Hitachi S-4800 microscope equipped with a cold field emission gun. For imaging, 1.5 kV acceleration voltage and 4 mm working distance were set to display the image by using both upper and lower secondary electron detectors.

Scanning transmission electron microscopy measurements in combination with energy-dispersive X-ray spectroscopy (STEM-EDX) were performed on a Thermo Fisher Talos instrument. The microscope was equipped with a high brightness field emission gun (X-FEG) and 4 SDD EDX detectors resulting in a collection angle of 0.9 sr. The electron beam energy was 200 keV and the beam current ranged from 330 to 350 pA. The electron beam was scanned across the region of interest several times acquiring 200–400 frames, with short acquisition times ranging from 10 to 50 μs per pixel to keep the dose rate low. The signal of all frames was integrated later to reach sufficient signal-to-noise ratio. To compensate for sample drift during EDS acquisition, Velox drift correction was applied by cross-correlation after each frame. Particle size distribution was estimated with ImageJ software.

STEM in combination with electron energy loss spectrometry (STEM-EELS) has been recorded in a double-corrected Jeol JEM-ARM200F equipped with a cold field emission gun, operated at 200 keV, and a Gatan GIF Quantum. EEL spectra were recorded with a semicollection angle of 20.1 mrad in a 24 × 24 pixel spectrum image with a step size of 150 pm and pixel time of 0.25 s. Spatial drift correction was applied after each scanning row. The spectra were recorded in dual EELS mode, where the low-loss spectrum was used for energy drift correction and deconvolution of the according high-loss spectrum including the Fe-L_3,2_ edge.

Temperature-programmed reduction in hydrogen (TPR-H_2_) was conducted in a fixed-bed quartz reactor using 50 mg of catalyst. The catalysts were treated in Ar (100 mL·min^–1^) at 100 °C for 30 min before the reduction treatment. Subsequently, the materials were heat-treated up to 900 °C in 5% H_2_/Ar (80 mL·min^–1^, heating rate: 5 °C·min^–1^), and the H_2_ consumption was monitored with a thermal conductivity detector. The detector was calibrated with certified calibration gas mixtures and controlled with a CuO standard measurement.

X-ray photoelectron spectroscopy (XPS) was carried out in a SPECS spectrometer equipped with a Phoibos 150 MCD-9 detector. The analyses were conducted under ultrahigh vacuum (10^–9^ mbar) using nonmonochromatic Al K_α_ source (1486.6 eV) at an analyzer pass energy of 30 eV and an X-ray power of 100 W. Data treatment was carried out with the CasaXPS software after Shirley-type background subtraction. All the signals were referenced to the C 1s peak at 284.5 eV.

Diffuse reflectance infrared Fourier transform spectroscopy (DRIFTS) measurements were conducted using an Agilent Cary 680 FTIR spectrometer equipped with a MCT detector and a Harrick Praying Mantis diffuse reflection accessory DRP. The spectra were recorded at a spectral resolution of 1 cm^–1^ and 1024 scan accumulations at 40 °C. The catalysts were placed in an *in situ* cell (HVC-DRM-5 reaction chamber with a high pressure dome with ZnSe windows) and activated in H_2_ (Westfalen AG, 5.0) flow (50 mL·min^–1^) at 28 bar and at 200 °C (heating rate in flowing hydrogen 10 °C·min^–1^) for 2 h, followed by evacuation. After cooling down in vacuum to 40 °C, a spectrum was measured using the single beam of the spectrum of KBr as the background. Then, CO was dosed at increasing equilibrium pressures in the range between 0.05 and 45 mbar followed by evacuation to a pressure of 1 × 10^–5^ mbar. For these measurements, also the single beam of the spectrum of KBr was used as background. Difference spectra ((absorbance spectrum before CO adsorption) - (absorbance spectrum after CO adsorption)) were also calculated.

### Computational Methods

The structure and Raman spectrum of the ZnFe_2_O_4_ and Rh-doped materials were obtained with density-functional theory using the Vienna Ab Initio Simulation Package (version 5.4.4).^32,33^ The Perdew–Burke–Ernzerhof (PBE) functional was used to compute the exchange and correlation energy with a plane wave cutoff of 520 eV. Spin-polarized calculations were conducted to capture the magnetic nature of the materials. The DFT+U scheme^34^ was used with an effective Hubbard U parameter of 5.3 eV ascribed to Fe using the Dudarev method.^35^ The force convergence criterion in the geometry optimizations of the variable-length cubic unit cell was 0.02 eV/atom. From the optimized structures (8.53 Å unit-cell parameter), the normal modes were obtained via central finite differences with a 0.015 Å displacement and an energy convergence criterion of 1.0 × 10^–6^ eV.

### Catalytic Tests in the Hydroformylation of 1-Hexene

The catalytic tests were conducted in a 12 mL Teflon-lined stainless-steel reactor under magnetic stirring (750 rpm). The reactions were carried out in 1.5 mL toluene with the addition of 30 mg catalyst and 1.5 mmol of 1-hexene. First, the catalyst was activated at 200 °C in 28 bar of H_2_ for 2 h. Subsequently, the solvent was added to the reactor, which was then purged with N_2_ for 5 min. Finally, 1-hexene was added, and the reactor was pressurized with syngas (CO:H_2_, 1:1, *p*_*total*_ = 40 bar). Hydroformylation tests were carried out at temperatures in the 25–100 °C range, and monitored by gas chromatography using decane as external standard. The formulas used for the calculation of conversion, yield, selectivity and aldehyde linearity are provided in the Supporting Information (Supporting Note 1).

Reusability tests were conducted under the same reaction conditions. The catalysts were recovered by centrifugation, washed with toluene and acetone, and dried in vacuum prior the subsequent catalytic tests. Rh leaching studies were carried out by stopping the reaction after 1 h and filtering the reaction crude twice with a 0.45 μm mesh. The filtered reaction crude without solid catalyst was incorporated into a clean reactor to further monitor the reaction.

### Turnover Frequency Calculation

The calculation of turnover frequencies for the ZnFe_2–*x*_Rh_*x*_O_4_-derived and impregnated catalysts activated at 200 °C for 2 h in 28 bar of H_2_ during the hydroformylation of 1-hexene was performed according to the following protocol.

The total amount of Rh present in the catalysts was determined by ICP OES analysis of the fresh spinel catalyst precursors or the calcined impregnated catalyst. The absolute quantity of Rh in moles for 30 mg of catalyst is given in Table S1. The fraction of metallic rhodium Rh^0^ in the activated catalysts was calculated from the XPS spectra of the catalysts reduced for 2 h at 200 °C and a pressure of 28 bar, which corresponds to the pretreatment before catalysis. The number of surface metallic Rh atoms (Rh^0^) was then estimated by a combination of electron microscopy and titration of surface Rh atoms by CO adsorption using DRIFT spectroscopy after exactly the same pretreatment. At first, the percentage of surface Rh atoms was calculated based on electron microscopy following the methodology described by Umpierre et al.^36^ and using the particle size distribution of the metallic Rh^0^ particles for the activated Rh-3.0 sample. It is important to note that we have considered half of the atoms obtained by this method, since we are dealing with exsolved nanoparticles, anchored to a metal oxide support, and not with free metal spheres. The amount of exposed Rh sites in the reduced Rh-3.0 sample determined in this way was then linked to the area of the infrared band found in the DRIFT spectrum of adsorbed CO (full coverage, achieved at *p*_*co*_ = 22 mbar). For the integration of the DRIFT spectra, fitting was necessary to exclude the peak of overlapping hydride species (Figure S1), which was subtracted from the total area. In this way, a correlation between exposed Rh^0^ sites and the integrated area measured by DRIFTS of adsorbed CO was obtained. The exposed Rh^0^ sites on reduced Rh-0.6, Rh-1.5 and Rh/ZFO were then extrapolated from the Rh carbonyl peak areas in their respective DRIFT spectra of adsorbed CO recorded at full coverage using the factor determined with the sample Rh-3.0 (Table S1).

With these numbers of exposed Rh^0^ atoms, TON vs time plots were constructed for each reaction and each catalyst, and the TOF was calculated from the slope at short reaction times (where turnover frequencies are constant) (Figure S2).

## Results and Discussion

### Characterization of the ZnFe_2–*x*_Rh_*x*_O_4_ Host Materials

**Figure 1:**
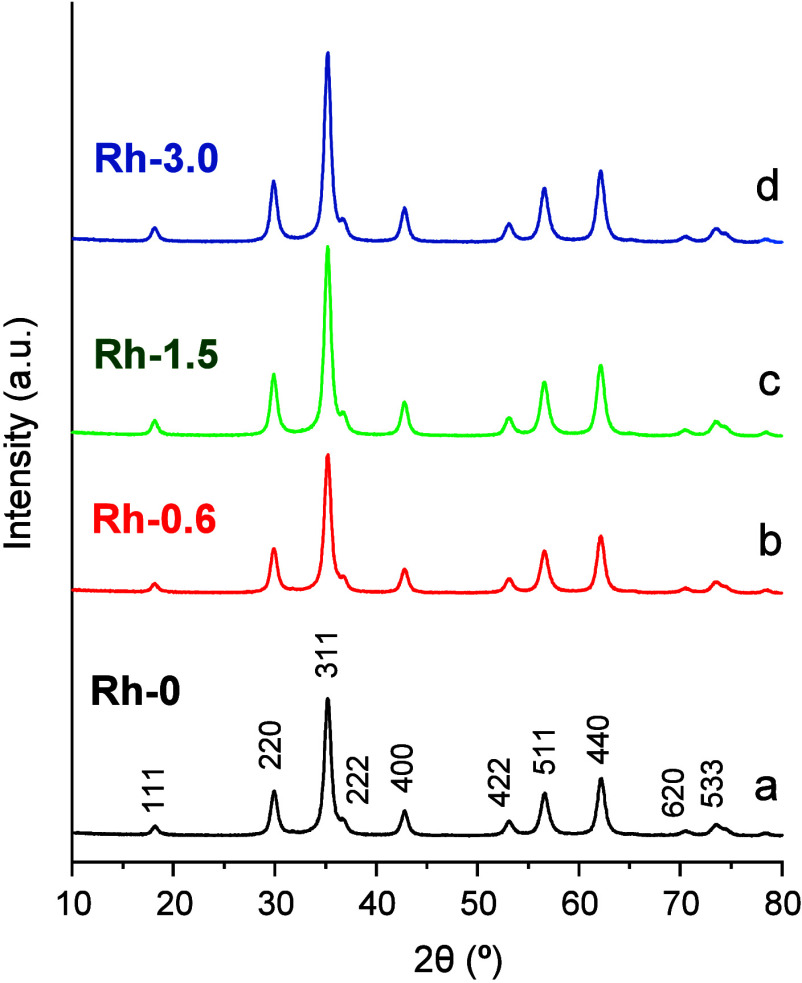
XRD patterns of the spinel catalyst precursors. (a) Rh-0; (b) Rh-0.6; (c) Rh-1.5; (d) Rh-3.0.

To ensure that rhodium is isomorphically incorporated into the oxide host structure during hydrothermal synthesis, the calcined spinels were analyzed in detail using complementary methods that capture the structure both integrally and locally, and with respect to bulk and surface. All the samples show diffraction peaks in the XRD patterns that can be assigned to a single-phase cubic spinel structure (space group *Fd*3̅*m*, ICSD: 170914) (Figure 1). The presence of Rh in the materials was confirmed by ICP OES, displaying a chemical composition which is consistent with that of the nominal content aimed at the synthesis (Table 1).

To further verify Rh incorporation into the spinel structure, the ZnFe_2–*x*_Rh_*x*_O_4_ precursors were analyzed by XRD profile fitting (Table 1, Figures 1 and S3). It can be seen that the calculated cubic cell parameters increase progressively with the amount of Rh incorporated (Table 1). According to these particular structural features, and the larger ionic size of Rh^3+^ with respect to Fe^3+^ (0.665 and 0.645 Å,^37^ respectively), this unit cell expansion could be ascribed to the isomorphic substitution of Rh^3+^ for Fe^3+^ species in octahedral sites of the spinel structure.

**Figure 2:**
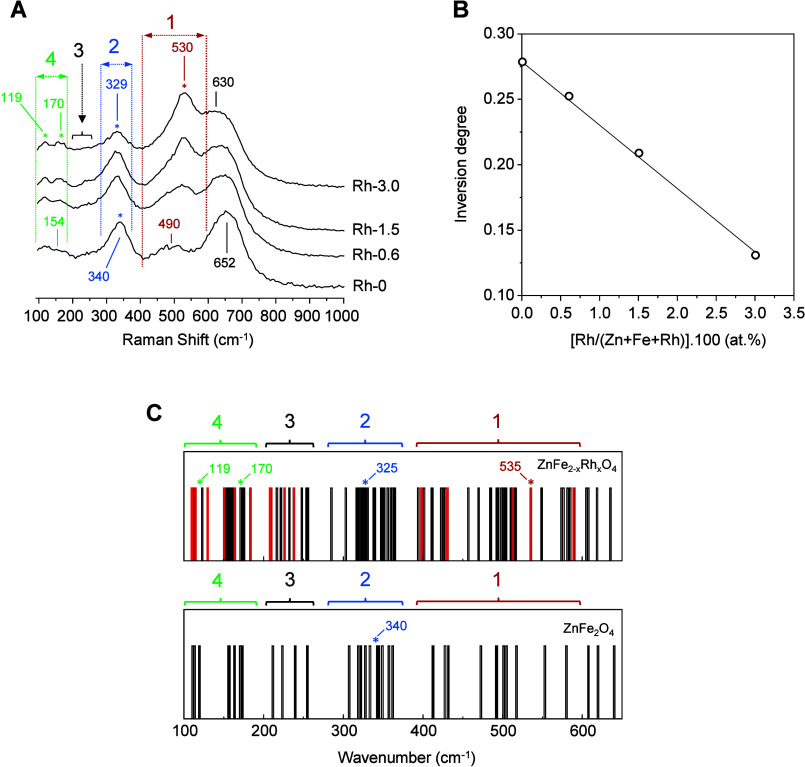
(A) Raman spectra of oxides with spinel structure measured at an excitation energy of 488 nm. (B) Inversion degree as a function of Rh content in Rh-0, Rh-0.6, Rh-1.5, and Rh-3.0 spinel oxides. (C) Vibrational normal-mode frequencies calculated by DFT for ZnFe_2_O_4_ and ZnFe_2–*x*_Rh_*x*_O_4_. Red bars indicate normal modes in which Rh^3+^ is significantly involved.

To exclude minority phases, the samples were analyzed by Raman spectroscopy (Figure 2). No additional Raman bands due to secondary phases were detected in the spectra. The undoped Rh-0 spinel displays Raman bands centered at 154, 340, 490, and 652 cm^–1^, which can be assigned to F_2g_ (1), F_2g_ (2), F_2g_ (3) and A_1g_ vibrational modes of the ZnFe_2_O_4_ spinel oxide structure (Figure 2A, Rh-0).^38^ The low frequency band F_2g_ (1) (154 cm^–1^) is generally attributed to translation modes in tetrahedral sites.^39,40^ On the other hand, the F_2g_ (2) (340 cm^–1^) signal can be assigned to M-O vibrations in octahedral sites of the spinel structure,^41^ although it has also been assigned to M-O vibrations in tetrahedral sites.^40^ In addition, the F_2g_ (3) band (490 cm^–1^) is assigned either to antisymmetric breathing,^42^ or asymmetric bending of M-O bonds in tetrahedral sites.^43^ The high frequency band A_1g_ (652 cm^–1^) is attributed exclusively to the symmetric breathing of M-O bonds in the tetrahedral sites in the spinel lattice.^42,43^

The incorporation of Rh leads to changes in the Raman profiles with respect to the undoped Rh-0 sample (Figure 2A, Rh-0.6, Rh-1.5 and Rh-3.0). On the one hand, the relative intensity of two peaks appearing at ca. 119 and 170 cm^–1^ increases. In parallel, the band at 340 cm^–1^ shifts to 329 cm^–1^. Additionally, the intensity of a new band centered at 530 cm^–1^ progressively increases with Rh content in the metal oxides, which has also been observed in other metal oxide systems after Rh-doping.^44^ Also, the peak at 652 cm^–1^ in Rh-0 shifts to lower frequencies, down to 630 cm^–1^ in sample Rh-3.0. This progressive shift to lower frequencies observed for the A_1g_ mode after the addition of Rh^3+^ can be interpreted in terms of changes in the inversion degree in the spinel structure.^45^

ZnFe_2_O_4_ is generally presented as a paradigmatic example of a normal spinel structure, in which M^3+^ species occupy octahedral sites, and M^2+^ cations are incorporated into tetrahedral sites of the crystal lattice.^46,47^ Although some degree of inversion could take place, by which part of Zn^2+^ would be incorporated into Fe^3+^ octahedral positions (with the subsequent incorporation of Fe^3+^ into tetrahedral sites), this effect has been reported to be relatively weak in the case of ZnFe_2_O_4_ spinels, and dependent on particle size and calcination temperatures.^47,48^

The A_1g_ mode can be fitted by two components centered at ca. 625 and 675 cm^–1^, assigned to Zn–O and Fe–O vibrations in tetrahedral positions, respectively (Figure S4).^49^ This makes it possible to estimate the degree of inversion in the corresponding materials by analyzing the relative intensity of these two peaks using the equation proposed by Nakagomi et al., modified for a normal spinel structure (Supporting Note 2, eq (6)).^45,49^ Deconvoluted Raman spectra, depicted in Figure S4, show a decrease in the relative intensity of the A_1g_ band at 675 cm^–1^ assigned to Fe–O vibrations in tetrahedral sites when the concentration of Rh increases. This means that the values for the degree of inversion estimated according to eq (6) in the Supporting Information show a progressive decrease from 0.27 for Rh-0, down to 0.12 for Rh-3.0 (Figure 2B). These inversion degree values are in agreement with those reported for ZnFe_2_O_4_ nanoparticles.^50^

With the purpose of gaining broad insight on the effect of the incorporation of Rh^3+^ in ZnFe_2_O_4_ on the corresponding Raman spectra, we have modeled the frequency of the normal vibrational modes in both undoped and Rh-doped ZnFe_2_O_4_ using DFT (Figure 2C). The Raman spectra were simulated considering the unit cell of the undoped ZnFe_2_O_4_ with a normal spinel structure, in which the central octahedral site is occupied by one Rh^3+^ cation (see Computational methods in the Methods section). Figure 2C compares the predicted frequencies of the lattice vibrations in ZnFe_2_O_4_ and Rh-doped ZnFe_2_O_4_. Calculations show an increase of the number of normal vibrational modes in the 100–640 cm^–1^ frequency range after the incorporation of Rh, which is due to the disappearance of the degeneration of several modes in the more symmetric undoped ZnFe_2_O_4_ (Tables S2 and S3). Interestingly, not only modes related to vibrations in which central RhO_6_ is significantly involved are generated after its incorporation (red bars in Figure 2C), but also additional lines in which Rh is not directly involved (black bars in Figure 2C). The changes predicted by theory can be linked to specific features in the experimental Raman spectra (Figure 2A). First, the increase in relative intensity of the band centered at 530 cm^–1^ could be explained by an increase in the density of normal modes in the 400–600 cm^–1^ frequency range after the incorporation of Rh (Figure 2A and C, zone 1). In addition, several vibrations in this region are predicted by the calculations, which mostly involve RhO_6_ octahedra (see red bars in Figure 2C). Particularly, the DFT calculations predict the formation of a new band at 535 cm^–1^, which can be assigned to bending vibrations in RhO_6_ octahedra (see Supplementary Data) in line with the experimental results. Besides, the shift of the Raman band at 340 cm^–1^ to lower frequencies (329 cm^–1^) (Figure 2A and C, zone 2) and the generation of two features at ca. 119 and 170 cm^–1^ (Figure 2A and C, zone 4) are also predicted by the models.

Despite this, discrepancies with theory can be found in the prediction of new signals in the 200–270 cm^–1^ range (Figure 2A and C, zone 3), where experimental Raman spectra do not show significant differences after the incorporation of Rh. This is likely due to the laser wavelength used in our experiments (488 nm), which systematically shows very low intensity bands for ZnFe_2_O_4_ in the 200–270 cm^–1^ region.^41^

**Figure 3:**
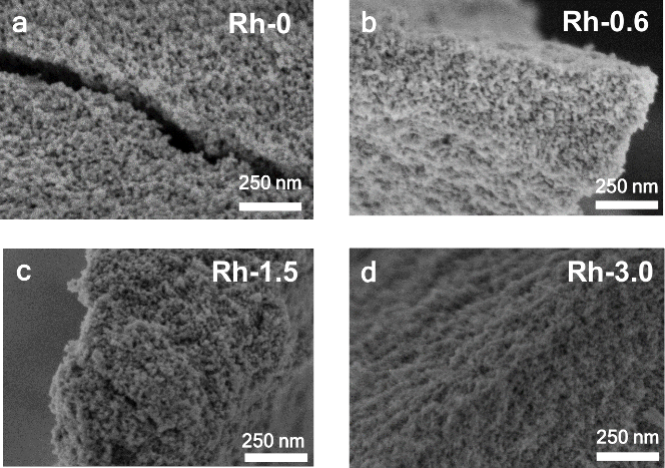
SEM images of the spinel catalyst precursors. (a) Rh-0; (b) Rh-0.6; (c) Rh-1.5; (d) Rh-3.0.

**Figure 4:**
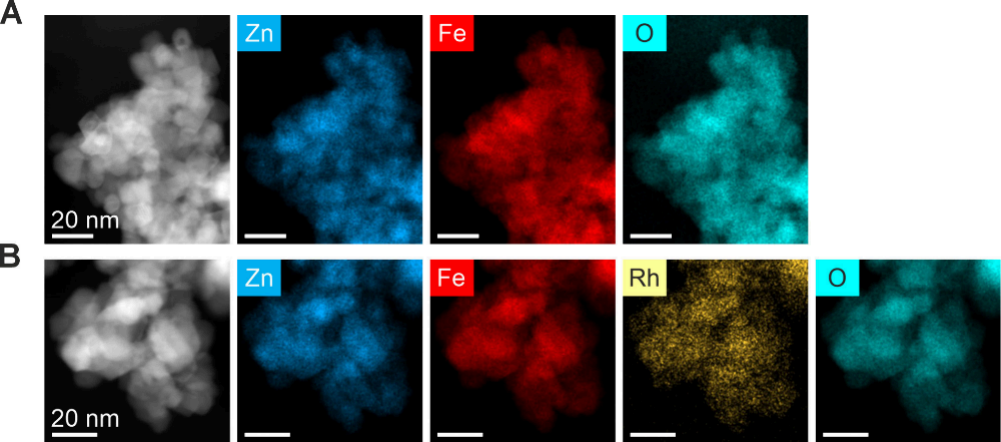
HAADF-STEM images and EDX maps of selected spinel catalyst precursors. (A) Rh-0; (B) Rh-0.6.

The nanostructure of the catalyst precursors was further analyzed by electron microscopy. SEM micrographs show the presence of nanoparticles of ca. 10–20 nm, which are aggregated in the form of agglomerates (Figure 3). These values agree with the crystallite size estimated from XRD patterns of the corresponding samples (Table 1), and with previous results reported for oxides with spinel structure synthesized in the presence of oxalate ions.^31^ HAADF-STEM images and their corresponding EDX maps for the synthesized spinel oxides **(**Figure 4 and Figure S5) reveal a high compositional homogeneity of the constituent elements in the metal oxide particles at this magnification.

The spinel-type oxides were also analyzed by N_2_-adsorption (Figure S6, Table 1). A progressive increase in the BET surface area from 43 m^2^·g^–1^ for Rh-0 up to 85 m^2^·g^–1^ for Rh-3.0 is observed when the Rh content increases (Table 1). In addition, N_2_-adsorption–desorption isotherms display a type IV hysteresis loop, which suggests the development of some degree of mesoporosity (Figure S6). Figure S7 shows the Barret-Joyner-Halenda (BJH) plots calculated from the corresponding desorption branch in the N_2_-adsorption–desorption isotherms shown in Figure S6. All the oxides exhibit a narrow pore size distribution, in which the pore size decreases concomitantly with the Rh content in the materials, from 13.3 nm for Rh-0 down to 7.4 nm for Rh-3.0 (Figure S7). According to these results and the microstructure observed by electron microscopy, the specific textural properties must derive from interparticle voids generated due to the agglomeration of fresh oxide nanoparticles. The changes in pore volume and diameter indicate that the presence of rhodium also influences the nucleation and growth processes under synthesis conditions.

In summary, it can be concluded that the synthesis yielded a series of phase-pure oxides. The proof of phase purity and the successful incorporation of rhodium is important for the following consideration of the exsolution of Rh particles from the spinel host structure by reductive treatment of the fresh oxides.

### Low Temperature Activation of ZnFe_2–*x*_Rh_*x*_O_4_ Precursors

**Figure 5:**
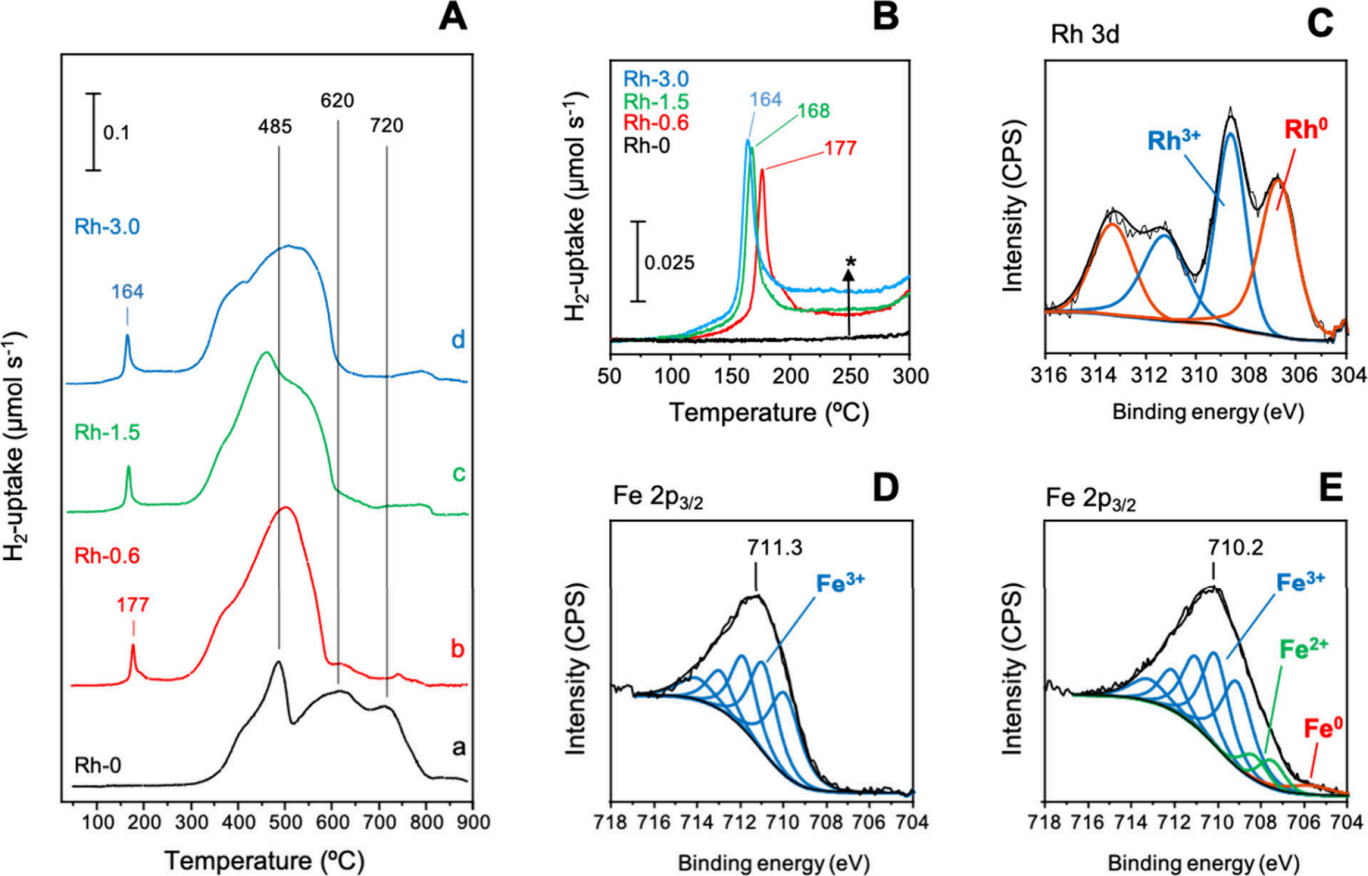
(A) TPR-H_2_ profiles of spinel catalyst precursors in the 50–900 °C range: (a) Rh-0; (b) Rh-0.6; (c) Rh-1.5; (d) Rh-3.0. (B) TPR-H_2_ profiles of spinel catalyst precursors in the 50–300 °C range. Experimental conditions: 5% H_2_/Ar, 80 mL min^–1^, 5 °C min^–1^. (C) Rh 3d core-level XPS spectra of the reduced Rh-3.0 sample. (D) Fe 2p_3/2_ core-level XPS spectra of fresh Rh-3.0. (E) Fe 2p_3/2_ core-level XPS spectra of reduced Rh-3.0. Reduction conditions: 28 bar H_2_, 200 °C for 2 h.

In order to investigate the reducibility of the host structures, the ZnFe_2–*x*_Rh_*x*_O_4_ metal oxide precursors were first analyzed by TPR-H_2_ in a wide temperature range (Figure 5). The hydrogen consumption profile of ZnFe_2_O_4_ displays three main reduction peaks at ca. 485, 620, and 720 °C (Figure 5A, profile a), which can be assigned to (i) the reduction of ZnFe_2_O_4_ to Fe_3_O_4_; (ii) the reduction of Fe_3_O_4_ to FeO and; (iii) the reduction of FeO to Fe, respectively.^51^ Incorporation of Rh^3+^ leads to the appearance of an additional reduction peak in the 150–180 °C temperature range, which can be assigned to the reduction of Rh^3+^ species to metallic Rh^0^ (Figure 5A, profiles b to d). In addition, the intensity of the main reduction peaks for undoped ZnFe_2_O_4_ at ca. 620 and 720 °C decrease drastically (Figure 5A, profiles b to d). It seems that the formation of metallic Rh could have an important influence on the reduction temperature of the spinel host structure. In this sense, the presence of Rh^0^ species at T ≥ 200 °C can favor H_2_ activation, thus aiding the reduction of the remaining oxidized species in the spinel oxide by hydrogen spillover, which thus occurs at lower temperatures.

Still, the ZnFe_2–*x*_Rh_*x*_O_4_ system exhibits two clear reduction regimes: (i) low-temperature reduction up to ca. 300 °C, in which Rh^3+^ species are reduced; (ii) high-temperature reduction in the 300–800 °C range, where the host structure is reduced and collapses. This feature allows to control Rh^3+^ reduction toward low temperature exsolution of Rh nanoparticles from ZnFe_2–*x*_Rh_*x*_O_4_ spinels while keeping the host structure intact. The limiting temperature here is 300 °C.

It should be noted that the total amount of H_2_ consumed in the experiments in the 200–800 °C range does not vary significantly with increasing Rh content (Table 1). However, two interesting features are observed when the low temperature reduction range (T = 160–180 °C) is analyzed in detail (Figure 5B): (i) a progressive shift of the peak for Rh reduction toward lower temperatures takes place at increasing Rh-contents and; (ii) H_2_ uptakes increase at temperatures just above the Rh^3+^ reduction peak (Figure 5B, see asterisk). These facts suggest that the presence of metallic Rh species favors the reduction of Rh^3+^ and part of Fe^3+^ species could be at least partially reduced at much lower temperatures. This spillover effect, by which additional species in the spinel structure are reduced (likely Fe^3+^), becomes more evident when quantifying the amount of Rh^0^ formed upon reduction from the TPR-H_2_ experiments (Figure S8 and Table S4). Experimental H_2_-uptake values for the first reduction peak (up to 211 °C) are higher than those expected from the nominal composition of the spinel oxide, considering the total reduction of only Rh^3+^ species, especially for low Rh contents. This indicates that part of Fe^3+^ must be reduced in order to explain the total H_2_-uptake for the first reduction peak in the TPR-H_2_ profiles.

This effect is further confirmed by the X-ray photoelectron spectra recorded after reduction at 200 °C in H_2_ (28 bar) for 2 h, resembling those activation conditions carried out before catalytic tests in hydroformylation (Figure 5C–E, Figure S9). The Rh 3d core-level XPS spectra of the metal oxide precursors show a peak centered at ca. 308.6 eV (Rh 3*d*_5/2_ peak), which can be assigned to the presence of surface Rh^3+^ species (Figure S9A, spectra a, c and e). After H_2_ treatment at 200 °C, a new peak at lower binding energy appears at ca. 306.7 eV, which is assigned to the formation of metallic Rh species (Figure 5C, Figure S9A, spectra b, d and f).^52^ The extent of Rh exsolution can be assessed by considering the surface Rh^0^/(Rh^0^+Rh^3+^) ratio obtained after H_2_ treatment, which increases from 0.35 for Rh-0.6 to 0.48 and 0.49 for Rh-1.5 and Rh-3.0, respectively (Table S1, Figure S9A). This indicates a less favored exsolution at low doping levels, a fact that has been observed in other metal oxide systems and ascribed to an enhanced interaction of the exsolvable metal cations with the host lattice at low metal loadings.^53^ The Fe 2p core-level XPS spectra of the oxides present a peak at 711.0–711.3 eV (Fe 2*p*_3/2_ peak), which agrees with the presence of surface Fe^3+^ species in zinc ferrite (Figure 5D, Figure S9B, spectra a, c and e).^54^ Subsequent activation under reducing conditions leads to (i) Fe 2*p* peak broadening and; (ii) a shift to ca. 710.2 eV for all the Rh containing samples (Figure 5E, Figure S9B, spectra b, d and f). This peak shift and broadening can be explained in terms of partial reduction of surface Fe^3+^ to Fe^2+^ and small amounts of Fe^0^ (Figure 5E).^54,55^ Considering Zn 2*p* core-level XPS spectra, both fresh (Figure S9C, spectra a, c and e) and activated (Figure S9C, spectra b, d and f) metal oxides samples display a peak centered at 1020.4–1020.6 eV (Zn 2p_3/2_), which can be ascribed to Zn^2+^ species in the spinel structure.^56^

**Figure 6:**
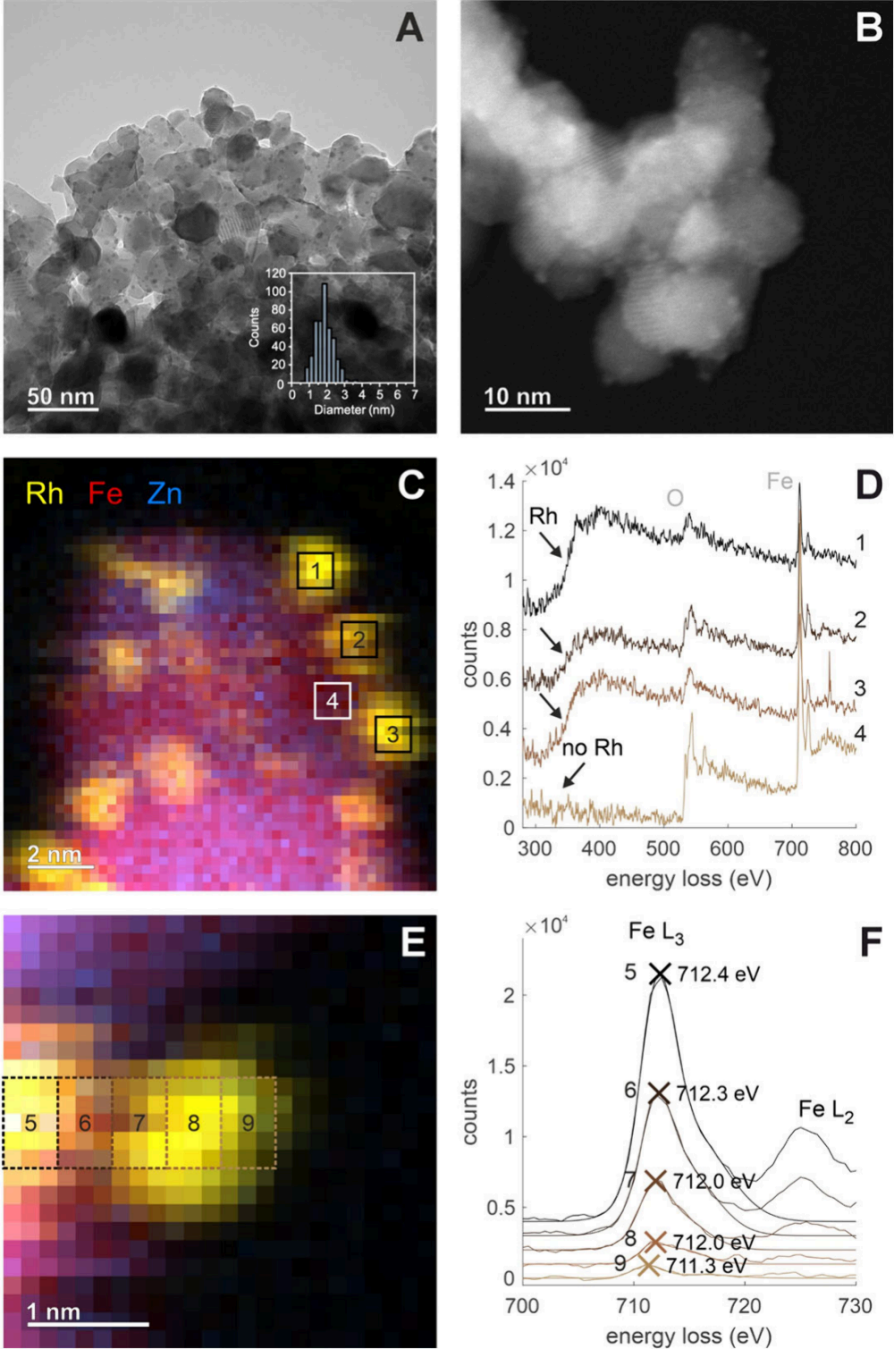
TEM images, EELS maps, and EELS spectra of the reduced Rh-3.0 sample. (A) BF-TEM image and Rh^0^ particle size distribution. (B) HAADF-STEM image. (C) EELS-map at medium magnification. (D) EELS spectra at the Rh-M_3_-, O K-, Fe L_3_- and L_2_-edges. (E) EELS map at high magnification. (F) EELS spectra at the Fe L_3_- and L_2_-edges. Spectra 1, 2, 3, and 4 and 5, 6, 7, 8, and 9 in D and F were collected in the corresponding areas marked in C and E, respectively.

To shed more light into the nature of metallic Rh species formed after low-temperature activation in H_2_, the Rh-3.0 sample was reduced under similar conditions (200 °C, H_2_, 28 bar for 2h) in a DRIFTS cell, and subsequently transferred to a TEM sample holder under inert conditions for further analysis by electron microscopy (Figure 6).

STEM images of the reduced Rh-3.0 sample display clear contrast differences, suggesting the formation of well-dispersed Rh metal nanoparticles socketed on the surface of the metal oxide particles, i.e., still partly embedded in the support (Figure S10), displaying a narrow particle size distribution centered at ca. 1.8 nm (Figure 6A and B). The chemical nature of these Rh nanoparticles and the surrounding support has been confirmed by conducting EELS spectrum imaging (Figure 6C to F). In accordance with exsolution, the reduced spinel material presents areas where the characteristic features of the Rh M_3_-edge are not observed (see spectrum 4 in Figure 6D, corresponding to area 4 in Figure 6C). Figure 6F displays the Fe L_3_- and L_2_-edge EEL spectra, corresponding to areas 5–9 indicated in Figure 6E. A shift toward lower energies, from 712.4 eV (Figure 6E and F, zone and spectrum 5) to 711.3 eV (Figure 6E and F, zone and spectrum 9) is observed when scanning from the bulk to the near-surface zone of the nanoparticle. This shift is consistent with the partial reduction of Fe^3+^.^57^ This result corroborates the XPS observation of a partial reduction of iron.

**Figure 7:**
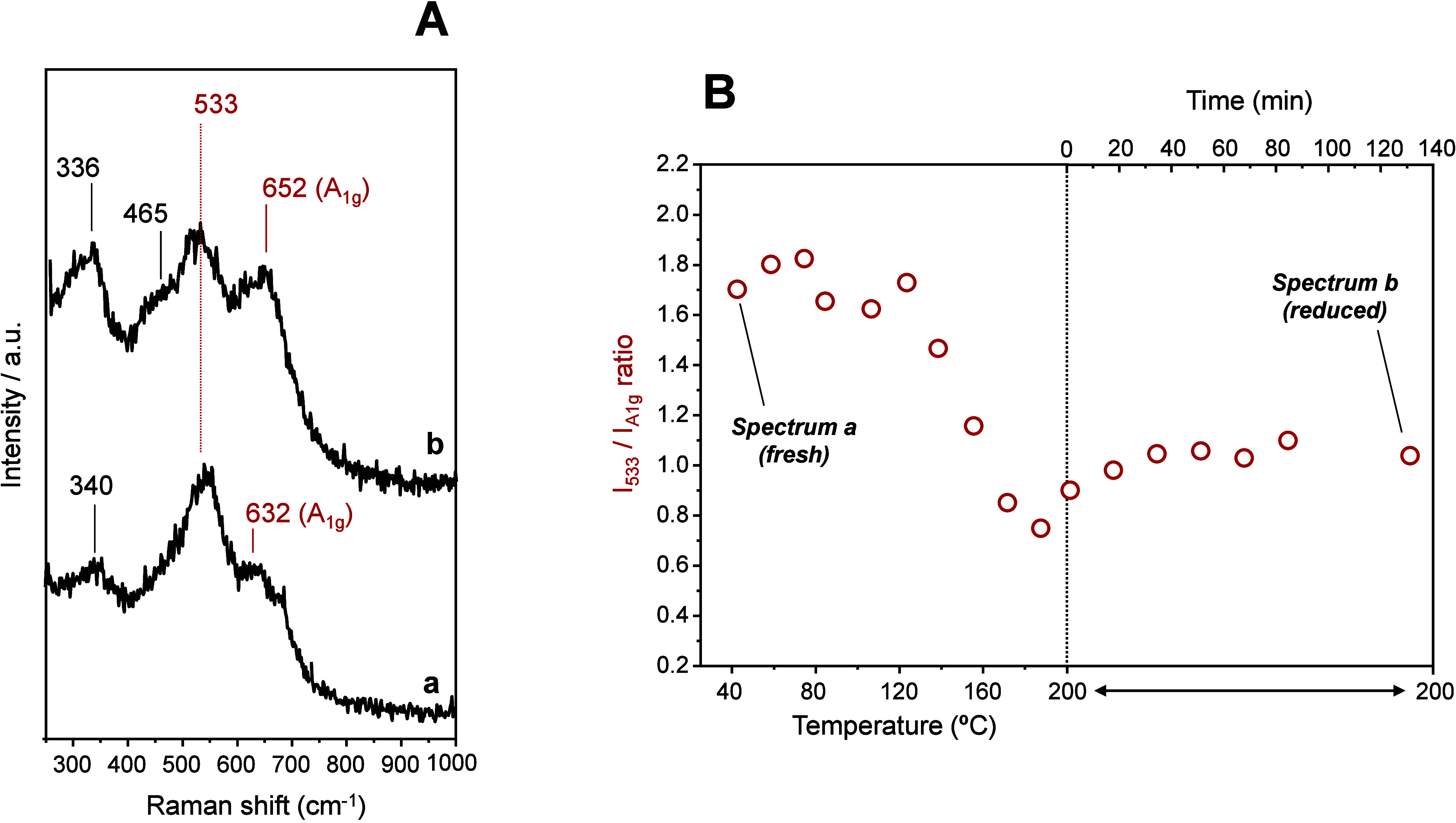
(A) Raman spectra of Rh-3.0 recorded before reduction treatment (a) and during reduction at 200 °C (5% H_2_, 2 h; b). (B) Evolution of the *I*_533cm-1_/*I*_A1g_ peak intensity ratio during reduction treatment at 200 °C in 5% H_2_ (30 mL·min^–1^) for 2 h (heating rate 10 °C·min^–1^).

With the aim of studying possible changes at the bulk level that would run parallel to Rh exsolution, Rh-3.0 was analyzed by *in situ* Raman spectroscopy during reduction at 200 °C in 5% H_2_ (1 bar) for 2 h. The spectrum prior to reduction (Figure 7A, spectrum a) shows differences in the relative intensities of the signals assigned to the spinel structure compared to the spectrum in H_2_ at 200 °C (Figure 7A, spectrum b), which suggest changes at the bulk level in the materials. Particularly, the relative intensity of the peak assigned based on *ex situ* Raman spectroscopy and DFT calculations (Figure 2) to Rh^3+^ species in the spinel structure (533 cm^–1^) decreases during reduction, as a consequence of the exsolution of Rh species from ZnFe_2–*x*_Rh_*x*_O_4_. In addition, the A_1g_ mode at 632 cm^–1^ shifts to higher frequencies, up to 652 cm^–1^ during reduction, which suggests also that the inversion degree of the metal oxide increases during the activation treatment. Interestingly, the spectral features evolve from those observed in Rh-doped spinels back to those found in the undoped ZnFe_2_O_4_ (Figure 2**A**, Rh-0). The evolution of the exsolution process was followed by recording the I_533 cm-1_/I_A1g_ intensity ratio as a function of temperature and time during the reduction process (Figure 7B). It can be observed that the decrease in the 533 cm^–1^ relative intensity starts at around 120 °C during the heating rate and continues its decreasing trend until 200 °C in agreement with the TPR results (Figure 5**A**). During the isothermal treatment for 2 h at 200 °C, there is no evidence of further changes at the bulk level, being the I_533 cm-1_/I_A1g_ intensity ratio stable. Both fresh and reduced precursors were analyzed by XRD diffraction, showing diffraction peaks corresponding to a spinel phase, with no other crystalline phases detected after reduction (Figure S11). The formed Rh nanoparticles were not observed in the XRD pattern due to the limitation of detecting such small Rh particles.

Based on these findings, we can draw the following conclusions on the low temperature exsolution process taking place during the activation of ZnFe_2–*x*_Rh_*x*_O_4_. First, Rh nanoparticles are formed from bulk Rh^3+^ species in the spinel structure. According to *in situ* Raman experiments, this process starts at ca. 120–140 °C (Figure 7B). The reduction treatment not only affects Rh^3+^, but also Fe^3+^ species. As evidenced by EEL spectroscopy, iron species in close proximity to the exsolved Rh nanoparticles seem to be partially reduced to Fe^2+^. In addition, small amounts of Fe^0^ were detected by XPS. In contrast, those Fe species which are not in contact with the Rh nanoparticles, more likely in the bulk of the spinel structure, are present as Fe^3+^. The combination of small Rh nanoparticles well-anchored to the small particle size oxide support in the activated materials can lead to interesting catalytic properties, especially in reactions prone to Rh leaching, like olefin hydroformylation. This is examined in the following.

### Catalytic Properties in Hydroformylation of 1-Hexene

**Scheme 1:**
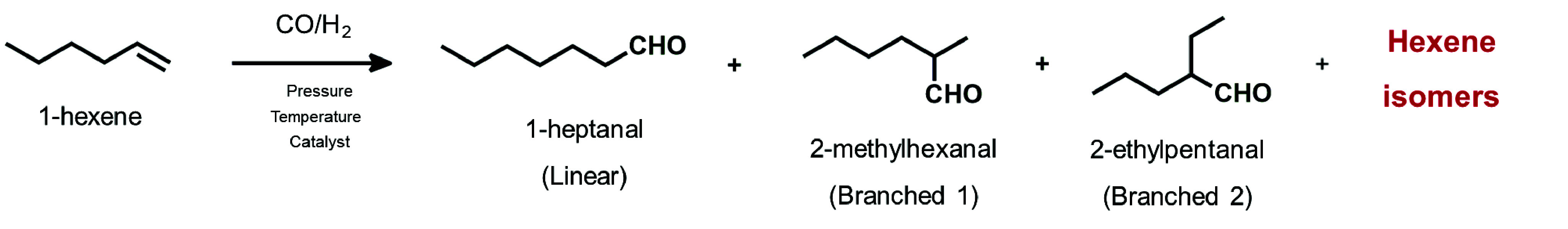
Hydroformylation of 1-Hexene Showing the Main Reaction Products

While straight-chain and branched aldehydes are the expected products of hydroformylation, isomers of 1-hexene can be expected as byproducts (Scheme 1). In the present study, the formation of alcohols and alkanes as hydrogenation products was not observed in any case.

**Figure 8:**
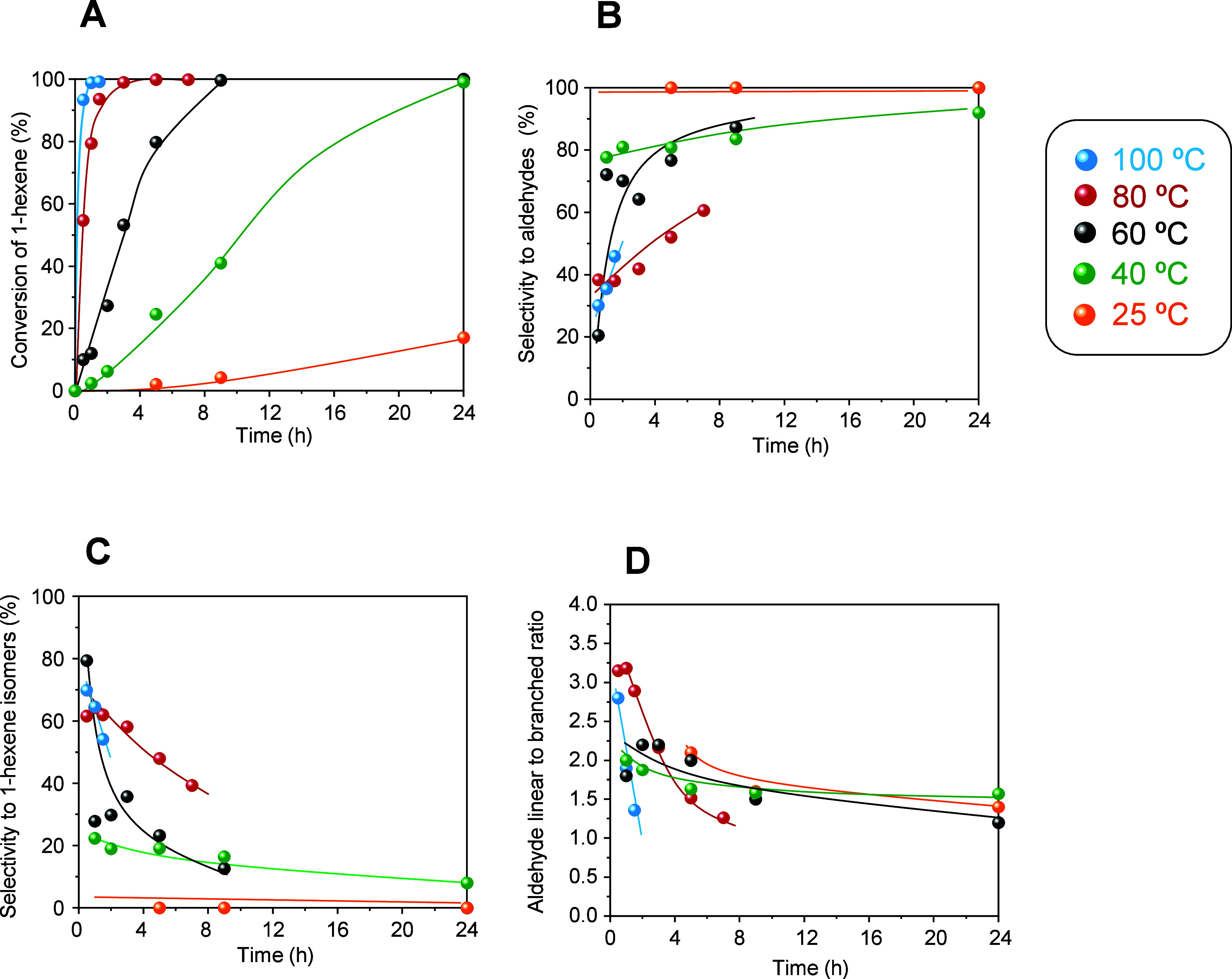
Catalytic properties in the hydroformylation of 1-hexene for Rh-3.0 in the 25–100 °C temperature range. (A) Conversion of 1-hexene. (B) Selectivity to aldehydes. (C) Selectivity to 1-hexene isomers. (D) Linear to branched aldehyde ratio. Reaction conditions: *m*_catalyst_ = 30 mg; 1.5 mmol of 1-hexene, 1.5 mL of toluene, *p* = 40 bar, H_2_/CO = 1:1. Red, blue, black, green, and orange symbols and lines correspond to the reaction temperature of 100, 80, 60, 40, and 25 °C, respectively.

The activated catalyst with the highest Rh content presents a notable activity at low reaction temperatures (25 and 40 °C, orange and green lines in Figure 8A, numerical values in Table S5). Increasing the temperature not only leads to a substantial increase of 1-hexene conversion (Figure 8A), but it also gives rise to changes in the product distribution (Figure 8B–D). The selectivity to aldehydes progressively decreases with reaction temperature (Figure 8B), in favor of the formation of 1-hexene isomers via isomerization (Figure 8C). Despite the drop of initial aldehyde selectivity at high reaction temperatures, the initial linear to branched aldehyde ratio is higher under high temperature reaction conditions (Figure 8D). However, the selectivity to linear products decreases with reaction time, especially at higher reaction temperatures (80 and 100 °C) from linear to branched ratios of ca. 3.0, down to ratios of ca. 1.5 due to the formation of branched aldehydes through the hydroformylation of the 1-hexene isomers (Figure 8D).

**Figure 9:**
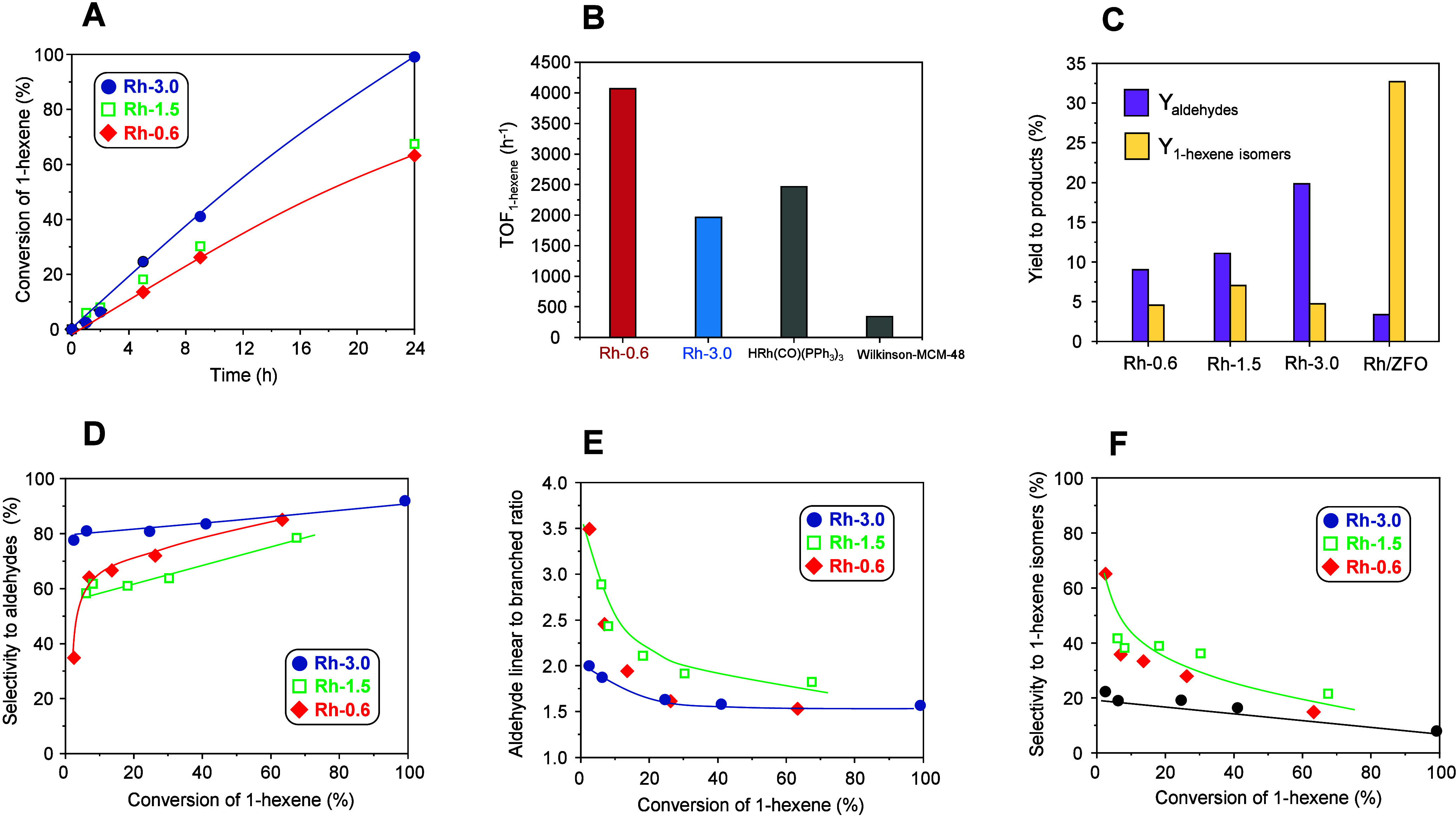
Catalytic properties of activated Rh-0.6 (rhombus), Rh-1.5 (hollow square), and Rh-3.0 (circle) during the hydroformylation of 1-hexene at 40 °C. (A) 1-Hexene conversion as a function of time. (B) Turnover frequency during the hydroformylation of 1-hexene at 100 °C for activated Rh-0–6 and Rh-3.0, compared with conventional homogeneous and supported Wilkinson catalyst.^60^ (C) Yield to main products recorded at 5 h of reaction for Rh-0.6, Rh-1.5, Rh-3.0, and supported Rh/ZFO. (D) Selectivity to aldehydes as a function of 1-hexene conversion. (E) Aldehyde linear to branched ratio as a function of 1-hexene conversion. (F) Selectivity to 1-hexene isomers as a function of 1-hexene conversion. Reaction conditions: *m*_catalyst_ = 30 mg; 1.5 mmol of 1-hexene, 1.5 mL of toluene, *p* = 40 bar, *T* = 40 °C, H_2_/CO = 1:1. Activation in 28 bar of H_2_ at 200 °C for 2 h prior to the catalytic tests.

The activity of the catalysts increases with increasing amount of Rh in the catalyst precursor (Figure 9A, numerical values in Table S6). On the other hand, at a reaction temperature of 100 °C, the highest turnover frequency for the consumption of 1-hexene was achieved on sample Rh-0.6 (4065 h^–1^), which is in the range of the most active materials reported for the hydroformylation of 1-hexene so far, like Rh_2_P or Rh-zeolite systems **(**Figure 9B, Table S7). The trend is also in line with reports about high efficiency of single-atom Rh catalysts in hydroformylation reactions.^58,59^

However, direct comparison of turnover frequencies with the literature is not straightforward because TOF values are strongly dependent on reaction conditions and how the turnover frequency is defined. The methodology for the calculation of both exposed Rh sites and TOF in the present study is described in the Methods section. Nevertheless, low Rh-content catalysts (Rh-0.6 and Rh-1.5) display a lower yield to aldehydes (Figure 9C). This is not only due to increasing activity with increasing Rh content in the catalyst precursor, i.e., with the presence of more Rh surface sites (Table S6), but also to the fact that catalysts with lower Rh content are more prone to olefin isomerization than hydroformylation compared to the Rh-3.0 catalyst (Figure 9D,F). Despite their lower selectivity, the linear to branched aldehyde ratios for low Rh content materials are higher, especially at low 1-hexene conversion (Figure 9E). For comparison, a 1 wt % Rh/ZnFe_2_O_4_ (Rh/ZFO) supported catalyst was synthesized by wet impregnation of ZnFe_2_O_4_, the basic characterization of which is summarized in Figure S12. The nominal metal content of this reference catalyst was selected so that it is in the range of the metal content of the spinel-derived catalysts, whereby it is directly comparable with Rh-0.6 (Table 1). The relatively low metal content was chosen to avoid the formation of large Rh particles, which are susceptible to leaching, given the comparatively small specific surface area of the ZnFe_2_O_4_ support of 43 m^2^·g^–1^. Indeed, small Rh particles in the range between 1 and 1.5 nm were obtained (Figure S12D), which are comparable to the particles on the spinel-derived catalysts, even for the exsolved catalyst with the highest content Rh-3.0 (Figure 6A). However, despite the high Rh dispersion in this catalyst, the Rh/ZFO supported catalyst shows high yields of 1-hexene isomers under the same reaction conditions (Figure 9C), in contrast to the catalysts obtained through exsolution. This indicates the unique functional properties of the Rh particles obtained by exsolution.

**Figure 10:**
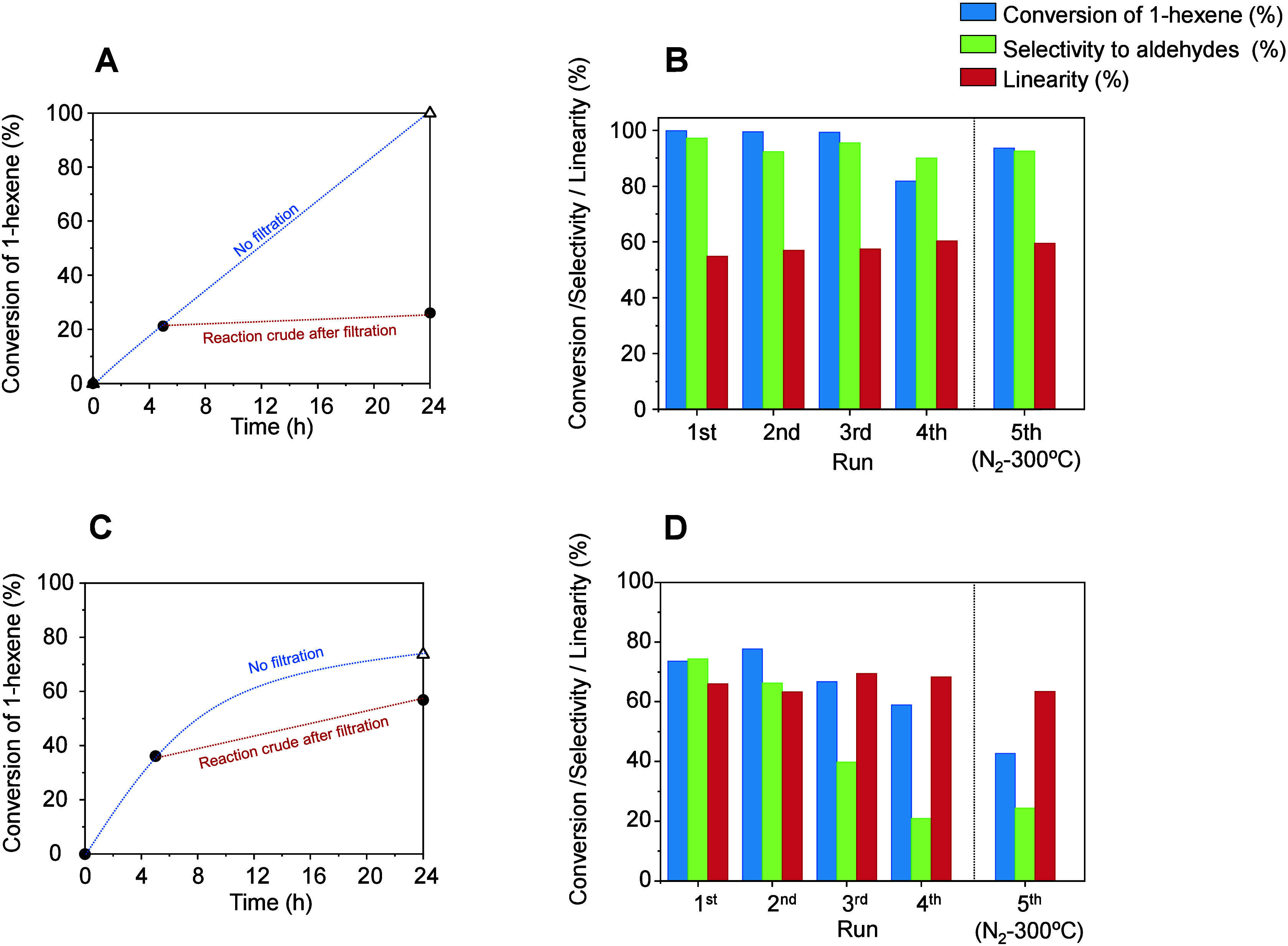
Hot filtration (A and C) and stability tests after five uses (B and D) on activated Rh-3.0 (A and B) and Rh/ZFO (C and D). Reaction conditions: *m*_catalyst_ = 30 mg; 1.5 mmol of 1-hexene, 1.5 mL of toluene, *p* = 40 bar, H_2_/CO = 1:1, *T* = 40 °C, 24 h. The fifth use was carried out after conducting a reactivation treatment under N_2_ flow (300 °C, 2 h). An activation treatment at 28 bar of H_2_ for 2 h was conducted prior to each use.

Selected spent catalysts were analyzed by XRD after the hydroformylation tests (Figure S13) confirming the stability of the host structure under the applied reaction conditions. Rh also does not appear to dissolve from the catalyst surface to a significant extent (Figure 10). Hot filtration tests on Rh-3.0 during hydroformylation reaction show a negligible 1-hexene conversion in the reaction mixture ca. 20 h after catalyst filtration, indicating no Rh leaching during the catalytic test (Figure 10A). In contrast to the catalyst obtained through exsolution, Rh/ZFO shows an increase in 1-hexene conversion after filtration (from 36.1 to 56.9%), indicating Rh leaching, which would be responsible for such a conversion increase in the absence of a solid catalyst (Figure 10C).

To confirm these differences in catalyst stability, a series of 5 consecutive tests in the hydroformylation of 1-hexene was carried out at 40 °C for 24 h on Rh-3.0 and Rh/ZFO (Figure 10B and D, respectively, numerical data in Table S8). The stability tests show that the activated Rh-3.0 catalyst retains its catalytic properties after the four uses, with only a slight drop in 1-hexene conversion (from 99.5 to 80.3%), while keeping both the selectivity to aldehydes and the linear to branched aldehyde ratio very stable (Figure 10B). Interestingly, the catalyst is regenerable, because it recovers its catalytic features in a fifth use, by carrying out a prior heat treatment in N_2_ (300 °C for 2 h). In sharp contrast, the supported Rh/ZFO catalyst suffers from a strong decrease in both 1-hexene conversion (from 73.6 to 42.7%) and selectivity to aldehydes (from 74.3 to 24.4%) after 4 uses (Figure 10D). In this case, a treatment at 300 °C in N_2_ for 2 h does not lead to a reactivation of the catalyst, but to a further decrease in the conversion of 1-hexene. These results agree with the Rh leaching observed in the hot filtration test in the case of the supported Rh/ZFO catalyst.

Finally, to underline the importance of the activation step to develop catalytic activity in ZnFe_2–*x*_Rh_*x*_O_4_ spinels, additional catalytic tests were conducted, by omitting the reduction stage in H_2_ for samples Rh-0.6 and Rh-3.0 at 100 °C (Table S9). Under these conditions, only Rh^3+^ species in the host oxide would be available initially for the reaction. In the absence of an activation step, sample Rh-0.6 shows no activity at similar reaction times as the activated precursor (98.5% 1-hexene conversion). The fresh Rh-3.0 catalyst has very low catalytic activity (28.9% 1-hexene conversion) compared to its reduced counterpart (99.2% 1-hexene conversion). These catalytic results indicate a long induction period to generate catalytic activity in the fresh spinel oxides, which can be ascribed to the slow formation of Rh^0^ on the surface under reaction conditions, thus excluding Rh^3+^ in the oxide as active species.

### The Nature of Surface Sites and Mechanistic Considerations

#### Metal Hydrides

**Figure 11:**
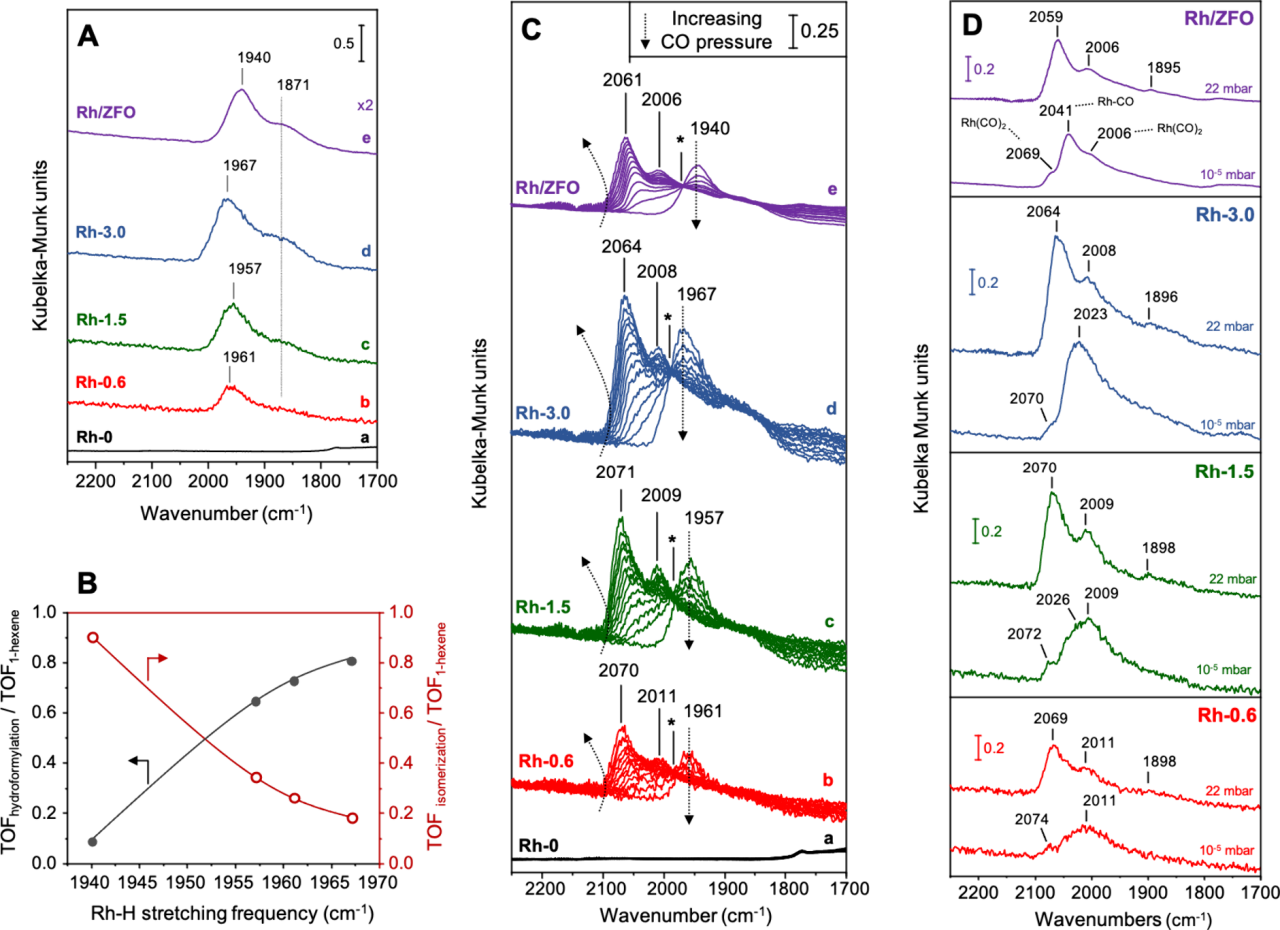
(A) DRIFT spectra recorded at 40 °C in the M–H_*x*_ stretching region (2250–1700 cm^–1^) of catalysts activated at 200 °C in H_2_ (28 bar) for 2 h and measured after evacuation and subsequent cooling down to 40 °C using KBr as background. (B) TOF_hydroformylation_/TOF_1-hexene_ and TOF_isomerization_/TOF_1-hexene_ ratios as a function of the Rh–H stretching frequency measured after activation at 200 °C in H_2_ (28 bar). Reaction conditions: *m*_catalyst_ = 30 mg; 1.5 mmol of 1-hexene, 1.5 mL of toluene, *T* = 40 °C, *p* = 40 bar, H_2_/CO = 1:1. (C) DRIFT spectra of adsorbed CO in the C–O and M–H stretching region recorded at 40 °C and increasing equilibrium pressures of CO (0–44 mbar, arrows indicate the development of the bands with increasing partial pressure of CO, asterisks indicate isosbestic points) for activated ZnFe_2–*x*_Rh_*x*_O_4_ spinels and supported Rh/ZFO using KBr as background. The corresponding difference spectra ((absorbance spectrum after CO adsorption) – (absorbance spectrum before CO adsorption)) are shown in Figure S15. (D) DRIFT spectra of adsorbed CO recorded at 40 °C and full CO coverage (*p*_*co*_ = 22 mbar) and in a vacuum (*p*_co_ = 10^–5^ mbar) using KBr as a background. The catalyst designations are indicated on the respective spectra.

To understand the specific catalytic performance of both exsolved and supported systems, fresh ZnFe_2–*x*_Rh_*x*_O_4_ spinels and the supported Rh/ZFO precursor were activated at 200 °C in H_2_ at 28 bar and analyzed by DRIFT spectroscopy at 40 °C (Figure 11). After activation in hydrogen, only Rh-containing catalysts show the formation of two bands due to adsorbed species, centered at 1871 and 1940–1961 cm^–1^ (Figure 11A, spectra b to e).

These bands can be assigned to surface metal hydride species formed during the activation in H_2_, which *a priori* are difficult to ascribe to specific M–H_*x*_ (M: Zn, Fe, Rh) surface complexes given the similar frequency values reported in literature for M–H_*x*_ species.^61−63^ The integrated peak areas increase proportionally with the Rh concentration in the samples (Figure S14). However, it cannot be excluded that hydrides are also formed with partially reduced Fe species, which is only formed in the presence of Rh during the reductive pretreatment due to hydrogen spillover (Figures 5–6). In particular, the low frequency signal at 1871 cm^–1^ appears for all activated Rh-containing catalysts, regardless of composition and synthesis procedure. Therefore, the band at 1940–1961 cm^–1^ is assigned to Rh–H_*x*_ species, while the band at 1871 cm^–1^ is more likely to be attributed to hydrides of the components of the host structure. Further evidence for this is given below. In addition, the high frequency band in the 1940–1967 cm^–1^ range shows a notably lower Rh–H stretching frequency for the supported catalyst (Figure 11A, spectrum e, 1940 cm^–1^) than for activated ZnFe_2–*x*_Rh_*x*_O_4_ spinels (Figure 11A, spectra b to d, 1957–1967 cm^–1^). These differences in terms of Rh–H stretching frequencies can be interpreted considering stronger surface Rh–H bonds in the catalysts derived from spinel metal oxides compared to the supported Rh/ZFO catalyst. In agreement with the catalytic results, this suggests differences in the electronic nature of the surface sites that form the hydrides.

**Scheme 2:**
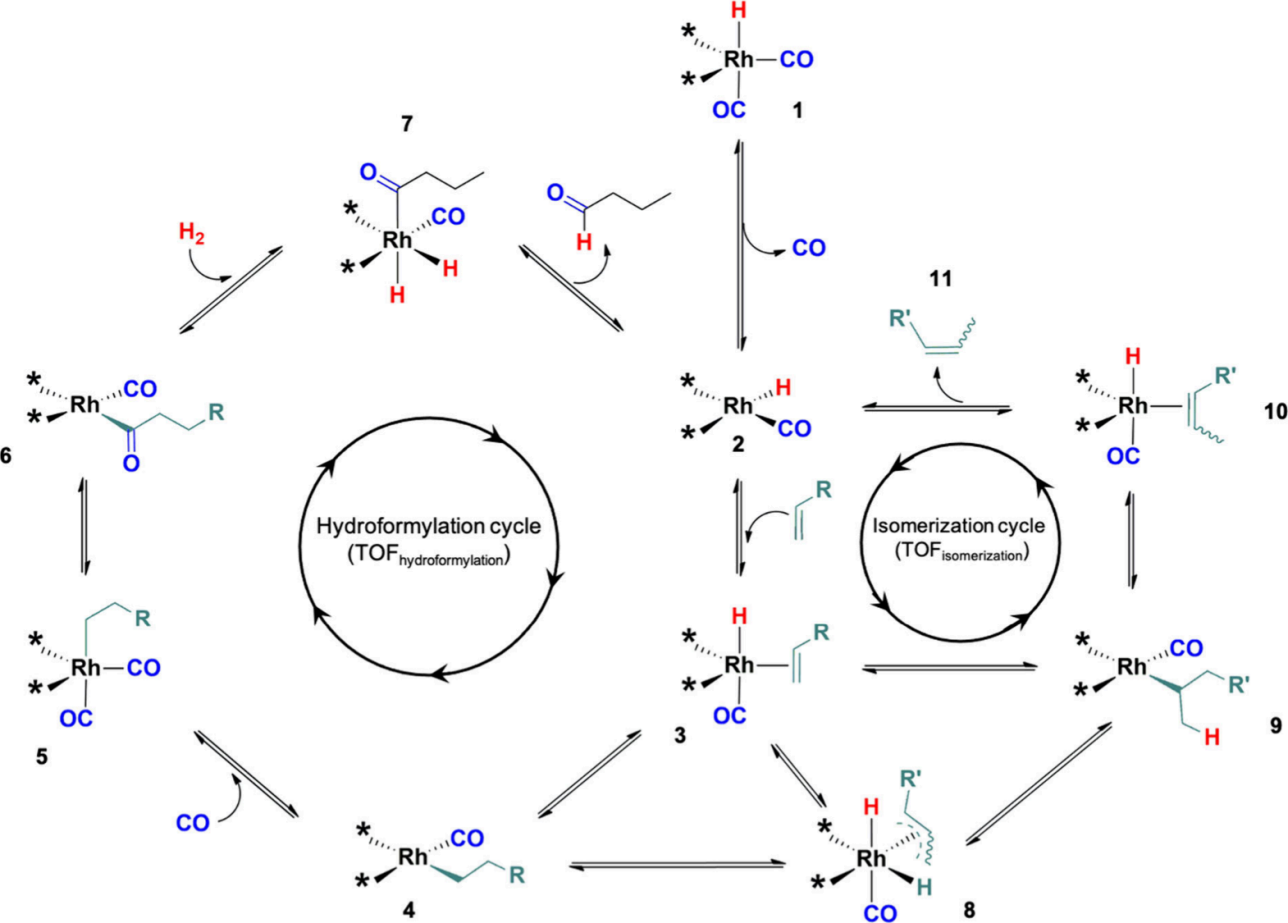
Coupled Olefin Hydroformylation and Isomerization Mechanisms

The strength of surface Rh–H bonds can have important consequences for the catalytic properties in the hydroformylation of 1-hexene. Scheme 2 depicts the combined mechanisms of both hydroformylation and olefin isomerization reactions for a hypothetical organometallic complex based on Rh anchored to the surface of the catalyst support. Hydroformylation works through the Heck-Breslow mechanism,^64^ which consists of: (1 → 2) release of CO to form an electron-deficient 16 e^–^ active state; (2 → 3) olefin coordination; (3 → 4) olefin insertion into the Rh–H bond via hydride migration; (4 → 5) CO addition; (5 → 6) formation of an acyl intermediate via nucleophilic attack of the alkyl group to the carbonyl and CO insertion into the Rh-alkyl bond; (6 → 7) oxidative addition of H_2_; (7 → 2) β-elimination to form the aldehyde product and restore the catalyst active state. It is important to note that after the olefin coordination step (Scheme 2, step 2 → 3), the reaction can follow an alternative pathway toward olefin isomerization (Scheme 2, step 3 → 8→9 → 10 → 11), which would run either via metal hydride transfer-β-elimination, or via π-allyl mechanisms.^65^ After isomerization, the formed olefin isomer can either be released as a product (Scheme 2, step 11) or it can be reincorporated into the Heck-Breslow hydroformylation cycle (Scheme 2, step 2 → 3) forming branched aldehydes. This way, the catalytic performance can be explained in terms of two coupled catalytic cycles running in parallel (i.e., hydroformylation and isomerization cycles). The specific surface features of each system will determine the preference for each of the possible reaction pathways.

Figure 11B presents the variation of the initial turnover frequency toward the formation of aldehydes and 1-hexene isomers with respect to the total turnover frequency (TOF_hydroformylation_/TOF_1-hexene_ and TOF_isomerization_/TOF_1-hexene_, respectively) as a function of the Rh–H stretching frequency measured by DRIFTS on activated ZnFe_2–*x*_Rh_*x*_O_4_ and supported Rh/ZFO catalysts. It can be seen that the initial TOF_hydroformylation_/TOF_1-hexene_ ratio increases concomitantly with the increase in Rh–H stretching frequency of surface hydrides. On the contrary, the opposite is observed for TOF_isomerization_/TOF_1-hexene_, displaying a decreasing trend at increasing Rh–H stretching frequencies. In other words, the strength of surface Rh–H bonds seems to determine the initial selectivity to aldehydes and 1-hexene isomers, showing a preference for the hydroformylation cycle (i.e., toward aldehyde formation) with respect to the isomerization reaction pathway for exsolved Rh nanoparticles, which display stronger Rh–H surface species.

The Heck-Breslow mechanism is complex, since most of the reaction steps are associated with similar energy barriers.^64^ Therefore, the nature of the rate-determining steps and the corresponding resting states is very sensitive to small changes in the reaction conditions or the nature of the catalyst. Despite this, when catalysts based on Rh-carbonyls are used (i.e., in the absence of bulky ligands, like phosphite or phosphine) it has been demonstrated that the oxidative addition of H_2_ is the rate-determining step for hydroformylation (Scheme 2, step 6 → 7).^66−70^ Hence, stronger Rh–H surface sites in exsolved Rh nanoparticles would favor H_2_ activation (Scheme 2, step 6 → 7) and increase the TOF_hydroformylation_ with respect to the TOF_isomerization_. On the other hand, the rate-determining step for olefin isomerization is the migratory insertion of the olefin into the Rh–H bond (Scheme 2, step 10 → 9).^71,72^ Based on this, weaker Rh–H bonds would favor isomerization, as in the case of the Rh/ZFO catalyst prepared by impregnation.

#### Surface Carbonyls

After activation in H_2_, the catalysts were exposed to increasing pressures of CO at room temperature, and the corresponding DRIFT spectra, which were recorded using KBr as background, are depicted in Figure 11C. As expected, only Rh-containing catalysts show the formation of new bands in the 2000–2080 cm^–1^ range due to surface-adsorbed CO (Figure 11C, spectra b to e), being these bands absent in the Rh-0 sample (Figure 11C, spectrum a). Interestingly, the intensity of the high frequency Rh–H stretching band (1940–1967 cm^–1^) decreases progressively at increasing CO pressures indicating ligand exchange. The development of the bands with increasing partial pressure of CO is indicated by the arrows in Figure 11C. This feature runs in parallel with the formation of two signals at 2006–2011 and 2061–2071 cm^–1^ in all Rh-containing materials, which can be ascribed to the asymmetric C–O stretching mode of Rh^δ+^(CO)_2_ gem-dicarbonyl species superimposed by the C–O stretching mode of linear Rh-CO adsorbed species in the range 2000–2060 cm^–1^, respectively (Figure 11C, spectra b to e).^73^ The presence of an isosbestic point at ca. 1990 cm^–1^ (see asterisks in Figure 11C) would suggest that CO substitutes hydride surface species at increasing CO pressures, with both CO and H coadsorbed on surface Rh sites. The coexistence of carbonyls and hydrides is an important prerequisite for catalytic activity. Conversely, the intensity of the hydride signal located at 1871 cm^–1^ does not vary after CO adsorption, confirming that it is likely due to Zn and/or Fe–H_*x*_ surface species formed after the activation (see also difference spectra in Figure S15), as proposed above.

To evaluate the stability of adsorbed CO species, the catalysts were outgassed at room temperature in the DRIFTS cell. Figure 11D displays DRIFT spectra of adsorbed CO in the C–O stretching region measured at full coverage (θ = 1, *p*_*co*_ = 22 mbar) and in high vacuum (*p*_*co*_ = 10^–5^ mbar). When analyzing the spectra recorded at full coverage, a new feature can be detected at ca. 1895–1898 cm^–1^, assigned to the C–O stretching mode of bridge Rh_2_(CO) species (Figure 11D). This feature is difficult to detect since it overlaps with the M-H signals. Under high vacuum, the intensity of the bands at ca. 2069–2074 cm^–1^ and ca. 2006–2011 cm^–1^ due to Rh^δ+^(CO)_2_ gem-dicarbonyl species decrease (Figure 11D), and the mode of linear Rh-CO at ca. 2041–2009 cm^–1^ becomes the dominating feature in the spectra. The C–O stretching modes are clearly shifted to lower wavenumbers on the metal particles formed by exsolution from ZnFe_2–*x*_Rh_*x*_O_4_, which can be attributed to a more pronounced π-backdonation from occupied Rh *d* orbitals to the antibonding π* orbital of the adsorbed CO.^74^ This suggests a more electron-rich nature of the metal particles generated by exsolution, which may also be related to partial alloy formation with iron. In addition, the donation of electrons from the 5σ orbital of the CO molecule to empty *d* orbitals of the metal is lower when CO is adsorbed on electron-rich metal particles. Since the 5σ orbital is weakly antibonding with respect to the C–O bond, reduced σ-donation can act in the same direction as increased π-backdonation, thus leading to an additional weakening of the C–O bond and to a red shift of the C–O stretching vibration signal. At the same time, less electron transfer from the 5σ orbital, located on the carbon atom of the CO molecule, to empty *d* orbitals of the metal is associated with a less strong C-metal bond, i.e., with weaker adsorption of the CO molecule on the metal particles. On this basis, the low C–O stretching frequencies also indicate a destabilization of the Rh–C bond of the adsorbed CO species on the surface of the ZnFe_2–*x*_Rh_*x*_O_4_-derived Rh particles. This is in line with a decrease in the intensity of the bands assigned to the C–O stretching modes when the catalysts are outgassed in the DRIFTS cell (Figure 11D). It can be seen that the extent of this decrease in intensity is higher for ZnFe_2–*x*_Rh_*x*_O_4_-derived catalysts, indicating that the lability of adsorbed CO on exsolved Rh nanoparticles is higher than in the case of supported Rh/ZFO.

All this would result in more labile surface CO adsorbed on well-anchored nanoparticles prepared by exsolution that would promote the nucleophilic attack of the alkyl intermediate to the carbonyl group to form the acyl-Rh intermediate in the hydroformylation cycle (see Heck-Breslow mechanism in Scheme 2, step 5 → 6), thus favoring the formation of aldehydes by hydroformylation.

In summary, the surface features of activated ZnFe_2–*x*_Rh_*x*_O_4_ and Rh/ZFO indicate marked differences in the nature of surface Rh sites depending on the synthesis procedure. The socketed nature of exsolved Rh nanoparticles (Figure S10) leads to a stronger metal–support interaction for catalysts derived from the low-temperature Rh exsolution from ZnFe_2–*x*_Rh_*x*_O_4_. The enhanced interaction between Rh and the spinel support not only gives rise to more stable catalysts for the liquid-phase hydroformylation of 1-hexene (showing no Rh leaching) with respect to supported Rh/ZFO, but also leads to (i) stronger Rh–H surface bonds that favor hydroformylation and prevent isomerization, and (ii) more labile CO surface species that promote hydroformylation. The low-temperature exsolution method thus represents a promising strategy for creating and designing novel interfaces between metal nanoparticles and oxide substrates, especially for low-temperature liquid-phase reactions. It is worth noting that a stronger metal-hydride bond, favored in the catalysts derived by exsolution due to a better metal–support interaction, would also decrease the hydricity and nucleophilicity of surface metal hydrides.^75^ Consequently, this effect leads to catalysts less prone to isomerization during the hydroformylation of 1-hexene, as observed in the case of exsolved Rh nanoparticles.

## Conclusions

The incorporation of Rh^3+^ into ZnFe_2_O_4_ spinel-type oxides results in phase-pure nanostructured materials capable of exsolving Rh nanoparticles at remarkably low temperatures, below 200 °C, in a hydrogen-containing atmosphere. Characterization of both fresh and reduced ZnFe_2–*x*_Rh_*x*_O_4_ precursors, using complementary experimental techniques and DFT calculations, revealed that these Rh nanoparticles form from bulk Rh^3+^ species under mild reduction conditions. The socketed nature of the exsolved metal particles on the surface of the host spinel enhances catalytic performance and provides high resistance to Rh leaching during the liquid-phase hydroformylation of 1-hexene. There is virtually no leaching of Rh from the catalysts prepared by low-temperature exsolution.

Insight into the role of the spinel support on the electronic and surface properties of the metal nanoparticles was gained through a combination of catalytic testing and surface analysis using DRIFTS surface analysis, which leverages the sensitivity of the adsorbed reacting molecules H_2_ and CO to the electronic structure of the adsorption centers on the surface of the metal particles. DRIFT spectroscopy confirmed that hydrides and carbonyls coexist on the surface of the catalysts at the same adsorption sites. A comparison between the surface species formed after activation and CO adsorption on Rh/ZnFe_2–*x*_Rh_*x*_O_4_ and a ZnFe_2_O_4_-supported Rh catalyst synthesized via wet impregnation revealed an enhanced electron transfer from the metal particles on the Rh-depleted spinel support to the adsorbed CO molecules resulting in more labile Rh-CO bonds. At the same time, higher Rh–H stretching frequencies are observed indicating stronger Rh–H bonds for the exsolved Rh nanoparticles.

These unique electronic and surface properties significantly influence the catalytic behavior during the liquid-phase hydroformylation of 1-hexene. Specifically, the stronger Rh–H surface interactions favor the hydroformylation pathway over isomerization. In addition, the increased lability of Rh-carbonyl surface species directs the reaction toward hydroformylation by promoting the formation of the acyl intermediate by nucleophilic attack of the alkyl group on the carbonyl, in accordance with the Heck-Breslow reaction mechanism.

These findings highlight the importance of advanced synthesis techniques for catalyst design, as they enable the tailoring of metal-promoter/support interaction to optimize catalytic performance in complex reactions. It is shown for the first time that the exsolution technique also offers significant advantages for low-temperature reactions at the solid–liquid interface in thermal catalysis.

## Supporting Information Available

Methods for calculating the catalytic parameters and the degree of inversion of the spinels; tables including frequencies of vibrational normal modes calculated by DFT for ZnFe_2–*x*_Rh_*x*_O_4_ and ZnFe_2_O_4_; tables with catalytic results in the hydroformylation of 1-hexene on activated and nonactivated ZnFe_2–*x*_Rh_*x*_O_4_ and Rh/ZnFe_2_O_4_ catalysts; additional figures with characterization results from fresh, reduced, and spent ZnFe_2–*x*_Rh_*x*_O_4_ and Rh/ZnFe_2_O_4_ catalysts (PDF)
Simulation of calculated vibrational modes I (MP4)
Simulation of calculated vibrational modes II (MP4)
