# Supplementary material for: Low-Temperature Exsolution of Rh from Mixed ZnFeRh Oxides toward Stable and Selective Catalysts in Liquid-Phase Hydroformylation
Source: J Am Chem Soc. 2025 Feb 10;147(7):5887–903. doi: 10.1021/jacs.4c14839 (PMC11848930; doi:10.1021/jacs.4c14839)
Supplement: Supplementary file 1 — ja4c14839_si_002.pdf [file ja4c14839_si_002.pdf]

Supporting Information for

Low-Temperature Exsolution of Rh from Mixed ZnFeRh Oxides towards Stable and Selective Catalysts in Liquid-Phase Hydroformylation

Daniel Delgado[1], Gregor Koch[1], Shan Jiang[1], Jinhu Dong,[1] Jutta Kröhnert[1], Franz-Philipp Schmidt[1], Thomas Lunkenbein[1], Carmen Galdeano Ruano[2], José Gaona-Miguélez[2], Diego Troya[3], Pascual Oña-Burgos[2], and Annette Trunschke[1*]*

[1]Department of Inorganic Chemistry, Fritz-Haber-Institut der Max-Planck-Gesellschaft, 14195 Berlin, Germany

[2]Instituto de Tecnología Química, Universitat Politècnica de València-CSIC, 46022 Valencia, Spain

[3]Department of Chemistry, Virginia Polytechnic Institute and State University, Blacksburg, VA, 24061, USA

*trunschke@fhi-berlin.mpg.de

*pasoabur@itq.upv.es

Supporting Notes

1. Determination of the catalytic properties in the hydroformylation of 1-hexene

The catalytic properties were calculated by using the following expressions, were n_i are initial moles, and n are moles detected during the reaction:

$$\text{Conversion} = \frac{n_{i\,1-hexene} - n_{1-hexene}}{n_{i\,1-hexene}} \cdot 100 \quad (1)$$

$$\text{Yield to aldehydes} = \frac{n_{aldehyde}}{n_{i\,1-hexene}} \cdot 100 \quad (2)$$

$$\text{Selectivity to aldehydes} = \frac{n_{aldehyde}}{n_{i\,1-hexene} - n_{1-hexene}} \cdot 100 \quad (3)$$

$$\text{Selectivity to 1} - \text{hexene isomers} = \frac{n_{hexene\ isomers}}{n_{i\,1-hexene} - n_{1-hexene}} \cdot 100 \quad (4)$$

$$\text{Linear to branched ratio} = \frac{n_{linear\ aldehyde}}{n_{branched\ aldehyde}} \quad (5)$$

2. Determination of the Inversion Degree from Raman Spectroscopy

$$x = \frac{I_{Fe}}{2(I_{M^{2+}} + R I_{Fe})} \quad (6)$$

The expression for the calculation of spinel inversion degree x (6) was modified for a normal spinel ferrite and adapted from references 1 and 2. $I_{M^{2+}}$ and I_{Fe} are the integrated areas of the contributions to the A_{1g} mode in the Raman spectra characteristic for Zn^{2+} (625 cm^{-1}) and Fe^{3+} (675 cm^{-1}), respectively. The R parameter represents the relative strength of the oscillator M^{2+}-O with respect to Fe^{3+}-O, assuming R=0.5 reported in referencences.[1-2]

Supporting Tables

Table S1. Amount of Rh^0 and exposed Rh^0 in 30 mg of catalyst after reduction for 2 hours at 200°C and a pressure of 28 bar

Sample	Total amount of Rh in 30 mg of calcined catalyst (moles)[a]	$Rh^0/(Rh^0+Rh^{3+})$ in the reduced catalyst[b]	Total amount of Rh^0 in 30 mg reduced catalyst (moles)	*D* (%)[c]	Integrated area of carbonyl bands measured by DRIFTS[d]	Total amount of surface Rh^0 (exposed Rh^0) in 30 mg of the reduced catalyst (moles)
Rh-0.6	$2.47581.10^{-6}$	0.35	$8.66533.10^{-7}$	n.d.	28.14	$6.04572.10^{-7}$
Rh-1.5	$4.93874.10^{-6}$	0.49	$2.41998.10^{-6}$	n.d.	56.38	$1.21129.10^{-6}$
Rh-3.0	$1.09989.10^{-5}$	0.48	$5.27949.10^{-6}$	27	66.39	$1.42639.10^{-6}$
Rh/ZFO	$2.88844.10^{-6}$	n.d.	n.d.	n.d.	35.44	$7.61408.10^{-7}$

a. From ICP-OES analyses

b. From XPS spectra

c. Dispersion *D* of metallic rhodium determined by electron microscopy and calculated from the mean particle size following the procedure discussed in reference 3

d. The area of carbonyl bands was separated from an overlapping hydride band by fitting; For the spectra and the fit see Figure S1

Table S2. Frequency of vibrational normal modes in cm^{-1} for $ZnFe_{2-x}Rh_xO_4$ calculated by DFT

635.44	504.20	423.15	339.00	238.22	158.42
618.53	504.20	423.15	338.76	238.22	158.42
607.50	503.53	412.09	338.34	237.98	156.87
607.50	500.70	411.70	338.34	232.21	156.40
605.37	500.70	411.17	330.44	232.21	155.99
605.37	498.46	411.17	330.44	226.20	155.99
589.98	498.46	401.53	327.46	221.82	153.33
589.04	494.44	398.32	326.81	221.82	153.33
589.04	494.10	398.32	324.82	221.59	153.01
585.02	493.71	398.13	324.82	216.76	150.38
581.73	493.71	394.19	324.60	216.76	129.82
581.73	493.15	394.19	324.60	209.48	129.82
576.38	492.60	364.75	323.71	208.10	122.77
573.85	492.60	364.75	320.90	208.10	114.33
549.11	485.44	363.72	320.90	183.32	114.28
548.69	484.66	363.72	318.13	183.32	114.28
548.69	469.94	361.88	317.67	175.37	114.22
535.49	469.94	359.71	317.67	174.36	113.69
535.49	456.96	355.71	316.62	172.91	113.69
516.43	456.96	355.71	303.20	172.91	111.15
515.31	430.76	351.48	303.20	172.71	110.43
515.31	430.16	348.77	285.05	170.96	110.43
514.96	430.16	348.52	255.43	170.96	110.12
514.96	429.65	348.52	255.43	163.69	109.73
512.91	428.02	347.68	254.25	161.76	109.73
511.60	427.70	347.68	254.03	161.76	
511.60	427.70	339.62	248.14	160.28	
510.51	426.05	339.62	248.14	160.28	

Table S3. Frequency of vibrational normal modes in cm^{-1} for $ZnFe_2O_4$ calculated by DFT

640.18	505.13	412.57	333.50	239.71	157.04
619.79	501.37	412.57	333.50	239.71	157.04
607.69	501.37	412.57	333.50	239.71	157.04
607.69	501.37	412.57	333.50	239.71	157.04
607.69	492.75	412.57	328.00	239.71	155.81
607.69	492.75	412.21	328.00	239.71	155.81
607.69	492.75	361.91	328.00	223.55	155.81
607.69	492.75	361.91	328.00	223.55	155.81
580.25	492.75	361.91	328.00	223.55	155.81
580.25	492.75	361.91	328.00	223.55	155.81
580.25	492.23	361.91	322.24	223.55	119.42
580.25	492.23	361.91	322.24	223.55	119.42
580.25	492.23	357.41	322.24	211.58	119.42
580.25	492.23	357.41	322.24	211.58	113.39
552.85	492.23	357.41	322.24	173.56	113.39
552.85	492.23	357.41	322.24	173.56	113.39
552.85	472.69	357.41	318.71	173.56	113.39
517.55	472.69	357.41	318.71	173.56	113.39
517.55	431.95	349.12	318.71	173.56	113.39
517.55	431.95	349.12	307.23	173.56	110.93
517.55	431.95	349.12	307.23	170.79	110.93
517.55	427.68	344.89	307.23	170.79	110.93
517.55	427.68	344.89	254.89	170.79	110.93
505.13	427.68	344.89	254.89	163.42	110.93
505.13	427.68	342.67	254.89	163.42	110.93
505.13	427.68	342.67	254.89	163.42	
505.13	427.68	333.50	254.89	157.04	
505.13	412.57	333.50	254.89	157.04	

Table S4. Theoretical H_2 consumption for full Rh^{3+} reduction and experimental H_2 consumption for the first reduction peak in TPR-H_2 profiles for $ZnFe_{2-x}Rh_xO_4$ oxides

Catalyst	Theoretical H_2 consumption for total Rh^{3+} reduction ($mmol_{H2}\cdot g^{-1}$)	Experimental H_2 consumption up to 211 ºC ($mmol_{H2}\cdot g^{-1}$)
Rh-0	0	0
Rh-0.6	0.11	0.30
Rh-1.5	0.28	0.36
Rh-3.0	0.55	0.48

Table S5. Catalytic properties of activated Rh-3.0 in the liquid phase hydroformylation of 1-hexene[a]

Reaction temperature (ºC)	Reaction time (h)	1-hexene conversion (%)	Yield to linear aldehyde (%)	Yield to Branched 1 aldehyde (%)	Yield to Branched 2 aldehyde (%)	Selectivity to aldehydes (%)	Selectivity to 1-hexene isomers (%)	Aldehyde linearity (%)	Aldehyde linear to branched ratio (%)
100	0.5	93.4	20.7	7.4	0.0	30.1	69.9	73.7	2.8
	1.0	98.9	22.9	10.9	1.1	35.4	64.6	65.5	1.9
	1.5	99.2	26.2	16.2	3.1	45.9	54.1	57.6	1.4
	3.0	98.7	29.9	22.2	7.0	58.9	41.1	50.7	1.0
	5.0	98.6	31.5	27.9	10.4	70.9	29.1	45.2	0.8
	8.0	98.4	33.7	32.5	11.6	79.1	20.9	43.3	0.8
	20.0	99.5	35.6	36.6	12.3	84.9	15.1	42.2	0.7
80	0.5	54.7	15.9	5.1	0.0	38.4	61.6	75.9	3.2
	1.0	79.4	21.5	6.8	0.0	35.7	64.3	76.1	3.2
	1.5	93.6	26.4	9.1	0.0	38.0	62.0	74.3	2.9
	3.0	99.1	28.4	13.1	0.0	41.9	58.1	68.4	2.2
	5.0	99.9	31.3	17.8	2.9	52.0	48.0	60.3	1.5
	7.0	99.9	33.8	22.2	4.6	60.7	39.3	55.8	1.3
	20.0	99.9	34.1	33.1	11.1	78.4	21.6	43.6	0.8
60	0.5	10.1	2.1	0.0	0.0	20.3	79.4	100.0	---
	1.0	12.0	5.5	3.1	0.0	72.2	27.8	64.3	1.8
	2.0	27.4	13.1	6.1	0.0	70.2	29.8	68.3	2.2
	3.0	53.3	23.4	10.8	0.0	64.2	35.8	68.3	2.2
	5.0	79.8	40.7	20.5	0.1	76.7	23.3	66.5	2.0
	9.0	99.7	51.8	32.3	2.9	87.3	12.7	59.5	1.5
	24.0	100.0	53.7	40.0	6.3	100.0	0.0	53.7	1.2
40	1.0	2.4	1.2	0.6	0.0	77.7	22.3	66.7	2.0
	2.0	6.2	3.3	1.8	0.0	81.0	19.0	65.2	1.9
	5.0	24.6	12.3	7.5	0.0	80.8	19.2	62.0	1.6
	9.0	41.0	20.0	12.6	0.0	83.6	16.4	61.3	1.6
	24.0	99.1	53.1	33.9	4.2	92.0	8.0	61.1	1.6
25	5.0	2.1	1.4	0.7	0.0	100.0	0.0	67.3	2.1
	9.0	4.2	2.6	1.6	0.0	100.0	0.0	62.0	1.6
	24.0	17.1	10.1	7.0	0.0	100.0	0.0	58.9	1.4
	48.0	43.9	25.3	18.6	0.0	100.0	0.0	57.5	1.4

a. Reaction conditions: $m_{catalyst}$ = 30 mg; 1.5 mmol of 1-hexene, 1.5 mL of toluene, p = 40 bar, H_2/CO = 1:1; Activation at 200 ºC in H_2 (28 bar) for 2h

Table S6. Catalytic properties of activated $ZnFe_{2-x}Rh_xO_4$ (Rh-0.6, Rh-1.5, Rh-3.0) in the liquid phase hydroformylation of 1-hexene at 40 ºC[a]

Catalyst	Reaction time (h)	1-hexene conversion (%)	Yield to linear aldehyde (%)	Yield to Branched 1 aldehyde (%)	Yield to Branched 2 aldehyde (%)	Selectivity to aldehydes (%)	Selectivity to 1-hexene isomers (%)	Aldehyde linearity (%)	Aldehyde linear to branched ratio
Rh-3.0	1.0	2.4	1.2	0.6	0.0	77.7	22.3	66.7	2.0
	2.0	6.2	3.3	1.8	0.0	81.0	19.0	65.2	1.9
	5.0	24.6	12.3	7.5	0.0	80.8	19.2	62.0	1.6
	9.0	41.0	20.0	12.6	0.0	83.6	16.4	61.3	1.6
	24.0	99.1	53.1	33.9	4.2	92.0	8.0	61.1	1.6
Rh-1.5	1.0	6.0	2.6	0.9	0.0	58.3	41.7	74.3	2.9
	2.0	8.0	3.5	1.4	0.0	61.8	38.2	70.9	2.4
	5.0	18.1	7.5	3.6	0.0	61.1	38.9	67.9	2.1
	9.0	30.2	12.7	6.6	0.0	63.8	36.2	65.7	1.9
	24.0	67.5	34.2	17.0	1.8	78.5	21.5	64.6	1.8
Rh-0.6	1.0	2.5	0.7	0.2	0.0	34.9	65.1	77.7	3.5
	2.0	6.9	3.2	1.3	0.0	64.2	35.8	71.1	2.5
	5.0	13.6	6.0	2.8	0.2	66.7	33.3	66.0	1.9
	9.0	26.2	11.7	6.2	1.0	72.0	28.0	61.8	1.6
	24.0	63.2	32.6	15.9	5.3	85.1	14.9	60.5	1.5

a. Reaction conditions: $m_{catalyst}$ = 30 mg; 1.5 mmol of 1-hexene, 1.5 mL of toluene, p = 40 bar, T= 40 ºC, H_2/CO = 1:1; Activation at 200 ºC in H_2 (28 bar) for 2h

Table S7. Catalytic properties of exsolved Rh nanoparticles and reference Rh-based catalysts in the hydroformylation of 1-hexene

Catalyst	Rh:1-hexene ratio	Temperature (ºC)	Pressure (bar)	Solvent	Reaction time (h)	Conversion (%)	Selectivity to aldehydes (%)	Linear to branched ratio	TOF (h^{-1})	Reference
Rh-3.0	1:136	100	40	Toluene	5	98.6	70.9	0.8	1965	This work
		80	40	Toluene	5	99.9	52.0	1.5	1150	This work
		60	40	Toluene	5	79.8	76.6	2.0	137	This work
		40	40	Toluene	5	24.6	80.8	1.6	53	This work
		25	40	Toluene	5	2.1	100	2.1	4.9	This work
Rh-1.5	1:304	40	40	Toluene	5	18.1	68.1	2.1	40	This work
Rh-0.6	1:606	100	40	Toluene	5	98.9	65.4	0.8	4069	This work
		60	40	Toluene	6	72.3	71.6	2.2	362	This work
		40	40	Toluene	5	13.6	66.7	1.9	71	This work
1%Rh@C	1:500	60	40	Toluene	5	63	72	2.03	308	[4]
1%Rh_2P@C	1:500	60	40	Toluene	5	96	94	2.03	3422	[4]
1%Rh_2P@C	1:500	100	40	Toluene	5	100	94	0.69	5883	[4]
5%Rh_2P@C	1:500	60	40	Toluene	5	77	69	2.22	155	[4]
$HRh(CO)(PPh_3)_3$	n.r.[a]	100	20	Toluene	1.05	99.8	99.8	2.55	2467	[5]
Wilkinson-MCM-48	n.r.	100	20	Toluene	3.50	100	99.5	2.33	338	[5]
Rh@Y	n.r.	120	60	Toluene	0.17	91	100	1.2	6567	[6]
Rh@Y	n.r.	60	60	Toluene	4	98	100	1.2	105	[6]
Rh@Y	n.r.	55	60	Toluene	4	n.d.	n.d.	n.d.	66	[6]
Rh@Y	n.r.	50	60	Toluene	4	48	100	1.7	47	[6]
Rh@Y	n.r.	40	60	Toluene	4	29	100	1.7	22	[6]

a. not reported

Table S8. Catalytic properties of activated Rh-3.0 and Rh/ZFO in the hydroformylation of 1-hexene at 40 ºC after 5 uses (24 h)[a]

Catalyst	Use	Reaction time (h)	1-hexene conversion (%)	Yield to linear aldehyde (%)	Yield to Branched 1 aldehyde (%)	Yield to Branched 2 aldehyde (%)	Selectivity to aldehydes (%)	Selectivity to 1-hexene isomers (%)	Aldehyde linearity (%)	Aldehyde linear to branched ratio
Rh-3.0	1st	99.9	53.3	37.3	6.5	97.2	2.8	54.9	1,2:1	99.9
	2nd	99.5	52.3	38.0	1.5	92.3	7.7	57.0	1,3:1	99.5
	3rd	99.3	54.4	38.8	1.6	95.5	4.5	57.4	1,3:1	99.3
	4th	81.8	44.5	29.1	0.1	90.1	9.9	60.4	1,5:1	81.8
	5th	93.6	51.6	31.5	3.6	92.6	7.4	59.5	1,5:1	93.6
Rh/ZFO	1st	73.6	36.1	18.6	0.0	74.3	25.7	66.0	1.9	73.6
	2nd	77.7	32.6	18.9	0.0	66.3	33.7	63.3	1.7	77.7
	3rd	66.7	18.4	8.1	0.0	39.7	60.3	69.4	2.3	66.7
	4th	58.9	8.4	3.9	0.0	20.9	79.1	68.3	2.2	58.9
	5th	42.7	6.6	3.8	0.0	24.4	75.6	63.5	1.7	42.7

a. Reaction conditions: $m_{catalyst}$=30 mg; 1.5 mmol of 1-hexene, 1.5 mL of toluene, p = 40 bar, H_2/CO = 1:1, T=40 ºC, 24 h; The 5th run was carried out by conducting a reactivation treatment under N_2 flow (300 ºC, 2h) before the catalytic test; An activation treatment at 200 ºC in H_2 (28 bar) for 2h was conducted prior each run

Table S9. Comparison of the catalytic properties in the hydroformylation of 1-hexene[a] at 100 ºC between activated and non-activated catalysts

Catalyst	Activation treatment	Reaction time (h)	1-hexene conversion (%)	Selectivity to aldehydes (%)	Selectivity to 1-hexene isomers (%)	Aldehyde linearity (%)
Rh-3.0	H_2 (28 bar)	1.5	99.2	45.9	54.1	57.6
	No activation	2.0	28.9	32.8	67.2	55.0
Rh-0.6	H_2 (28 bar)	1.5	98.5	38.6	61.4	59.9
	No activation	2.0	0	0	0	0

a. Reaction conditions: $m_{catalyst}$ = 30 mg; 1.5 mmol of 1-hexene, 1.5 mL of toluene, p = 40 bar, T= 100 ºC, H_2/CO = 1:1

Supporting Figures

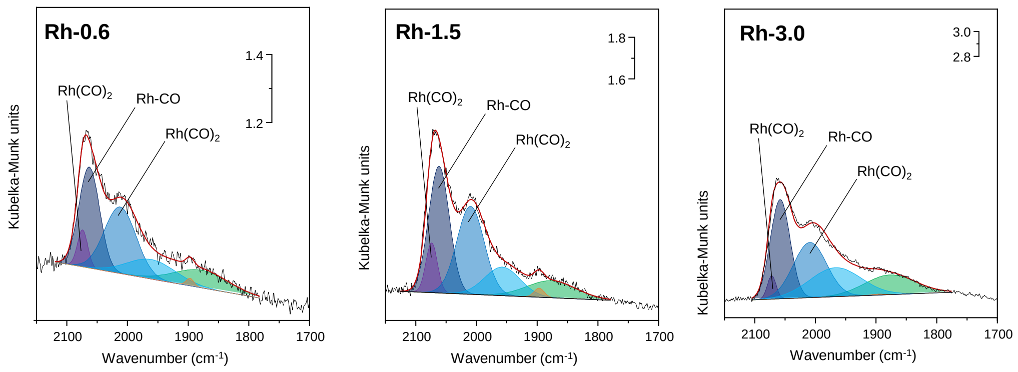

Figure S1. Room temperature DRIFT spectra of adsorbed CO recorded at full CO coverage (p_{CO}= 22 mbar) for reduced Rh-0.6, Rh-1.5 and Rh-3.0 catalysts. The peak indicated in green corresponds to residual metal hydride species and not to surface metal carbonyls (see **Figure 11 A** in the main text).

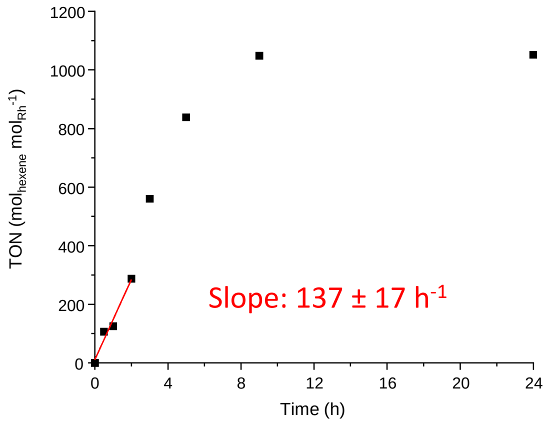

Figure S2. TON as a function of reaction time during the hydroformylation of 1-hexene at 60 °C on Rh-3.0. TOF is calculated from the slope at short reaction times. Reaction conditions*:* $m_{catalyst}$ =30 mg; 1.5 mmol of 1-hexene, 1.5 mL of toluene, p = 40 bar, H_2/CO = 1:1. Activation in 28 bar of H_2 at 200 °C for 2 h prior the catalytic tests.

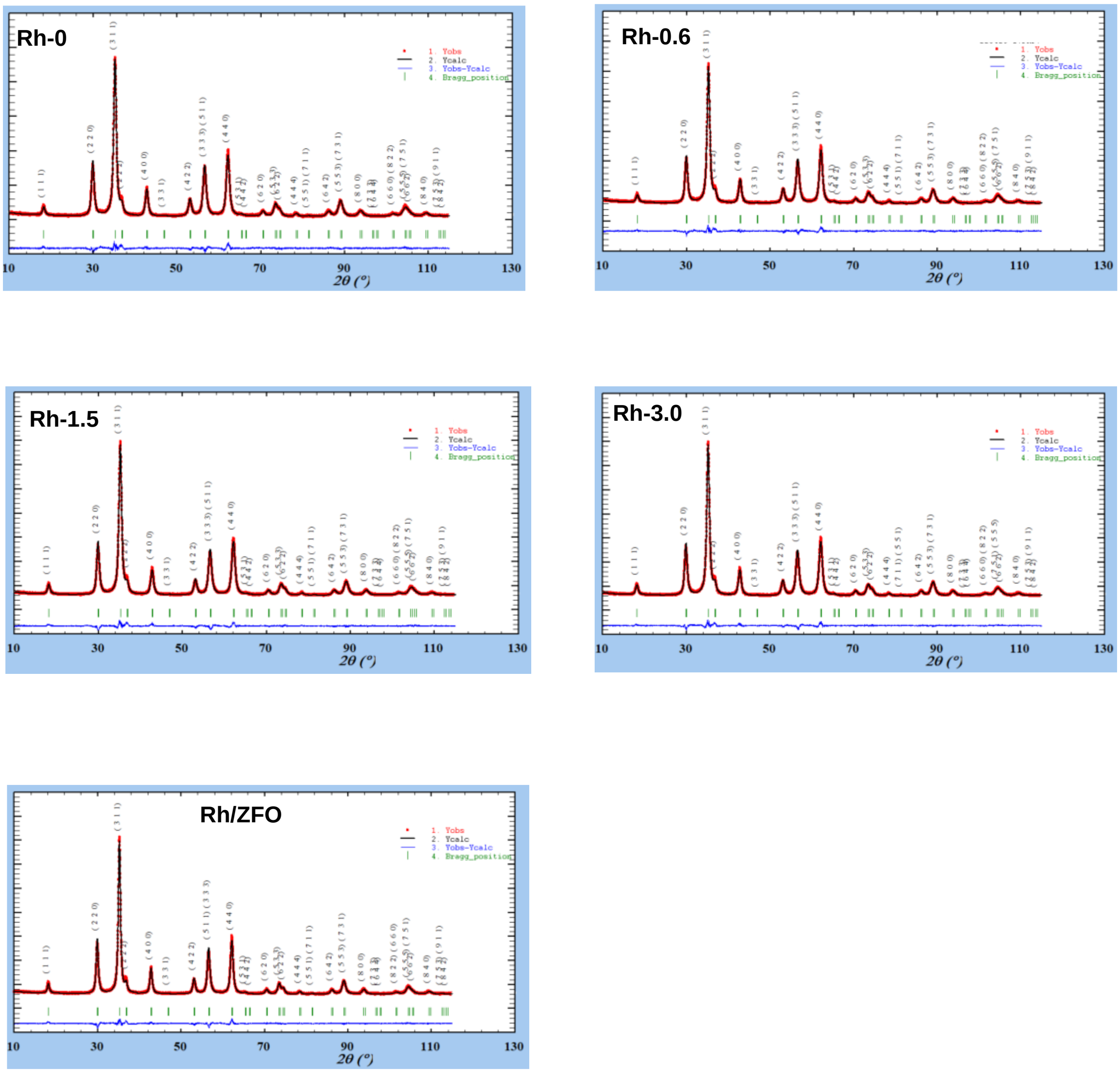

Figure S3. XRD Rietveld refinement plots of Rh-doped $ZnFe_2O_4$ catalyst precursors with a spinel structure.

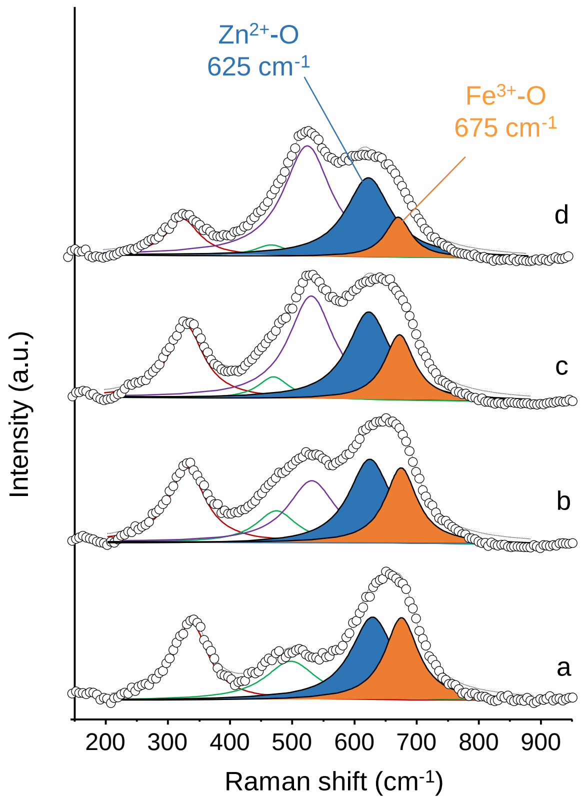

Figure S4. Deconvoluted Raman spectra of $ZnFe_{2-x}Rh_xO_4$ oxides. a) Rh-0; b) Rh-0.6; c) Rh-1.5; d) Rh-3.0.

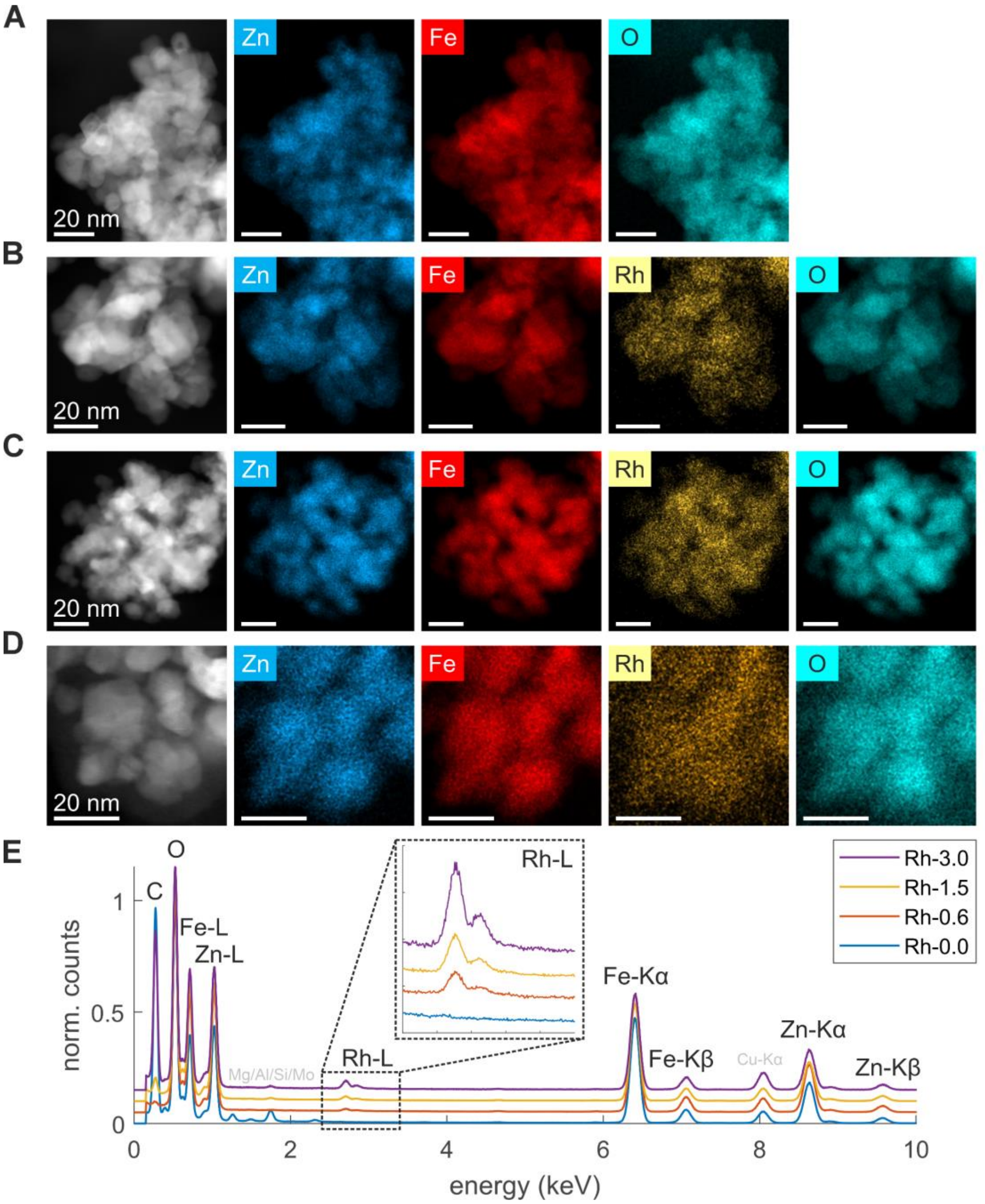

Figure S5. HAADF-STEM images and EDX maps of selected spinel catalyst precursors. A) Rh-0; B) Rh-0.6, C) Rh-1.5; D) Rh-3.0; E) Experimental EDX spectra.

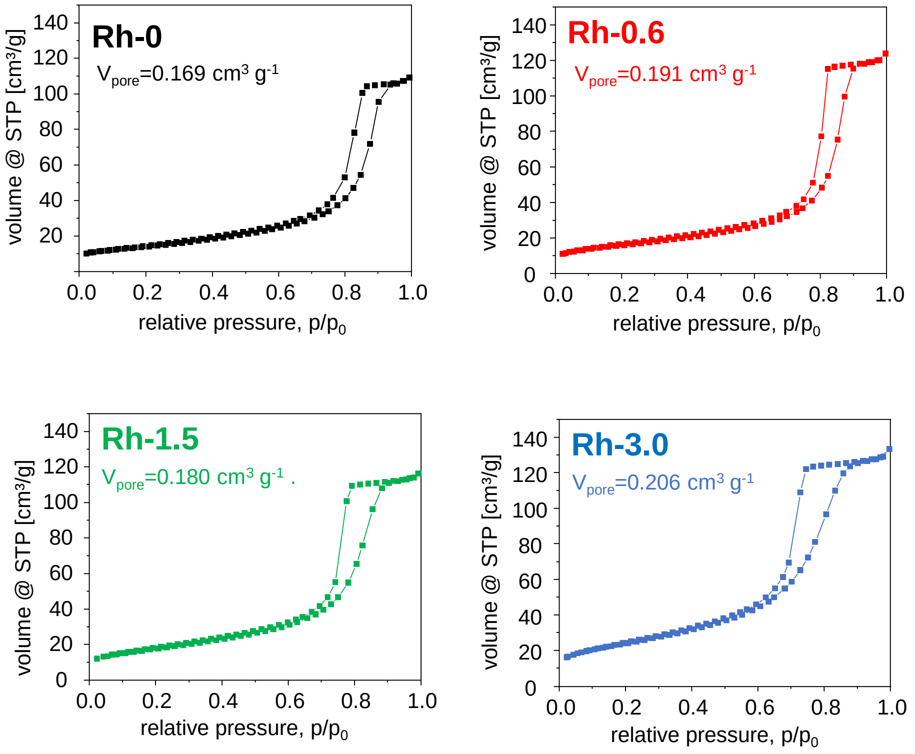

Figure S6. N_2-adsorption-desorption isotherms of Rh-doped $ZnFe_2O_4$ catalyst precursors. The total pore volume for each sample is indicated in the inset.

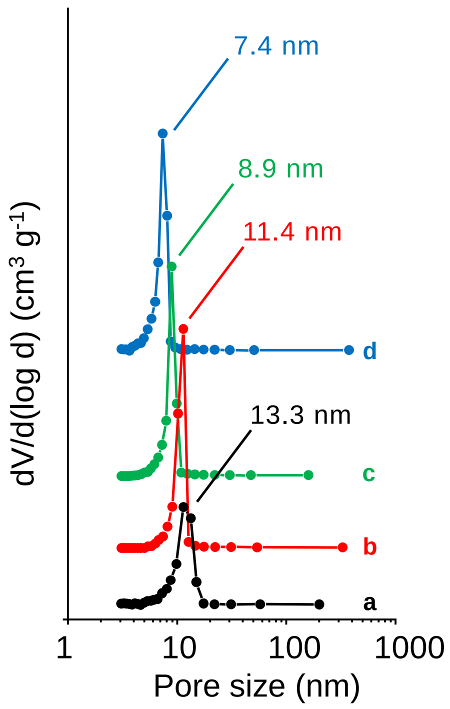

Figure S7. BJH plots for $ZnFe_{2-x}Rh_xO_4$ catalyst precursors. a) Rh-0; b) Rh-0.6; c) Rh-1.5; d) Rh-3.0. Note: plots calculated from the corresponding N_2-desorption branch shown in **Figure S6**. The numbers at the lines represent the pore diameter.

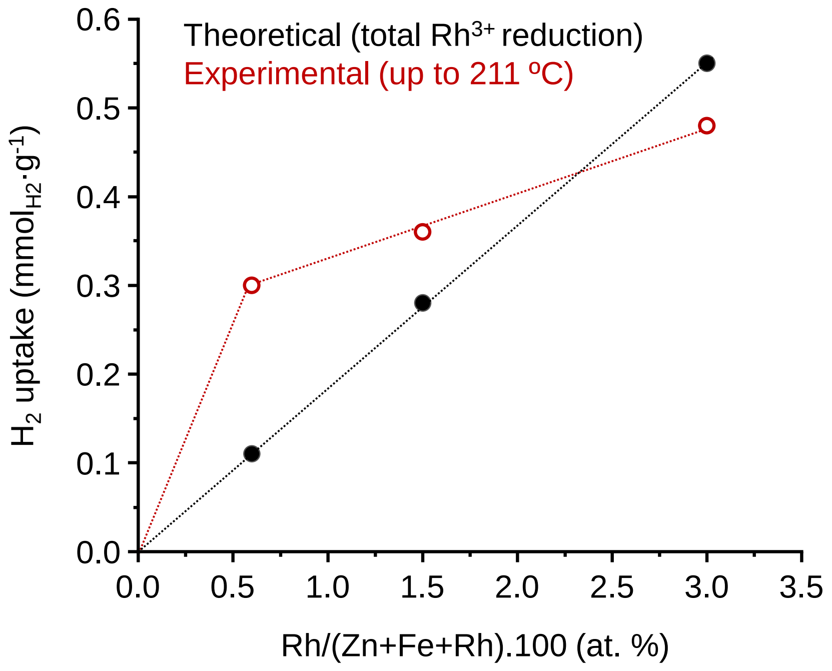

Figure S8. Theoretical (full Rh^{3+} to Rh^{0} reduction) and experimental H_2-uptake (first reduction peak in TPR-H_2) in temperature-programmed reduction at normal pressure (**Figure 5** in the main manuscript) as a function of the Rh content in the $ZnFe_{2-x}Rh_xO_4$ oxides.

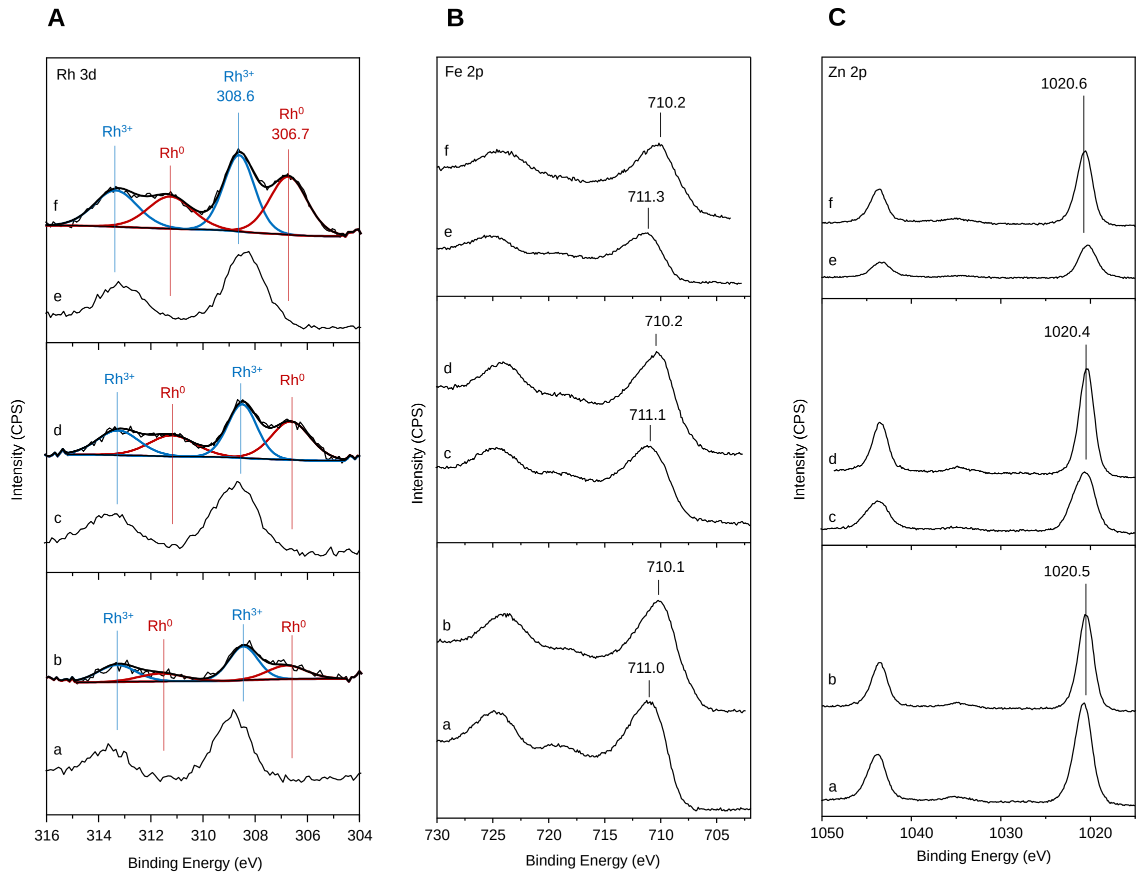

Figure S9. Rh 3d (A), Fe 2p (B) and Zn 2p (C) core-level XPS spectra of fresh Rh-containing metal oxide precursors $ZnFe_{2-x}Rh_xO_4$ (a, c and e) and activated catalysts (b, d and f); (a, b) Rh-0.6; (c, d) Rh-1.5; (e, f) Rh-3.0; Activation conditions: 28 bar H_2, 200 ºC for 2 h.

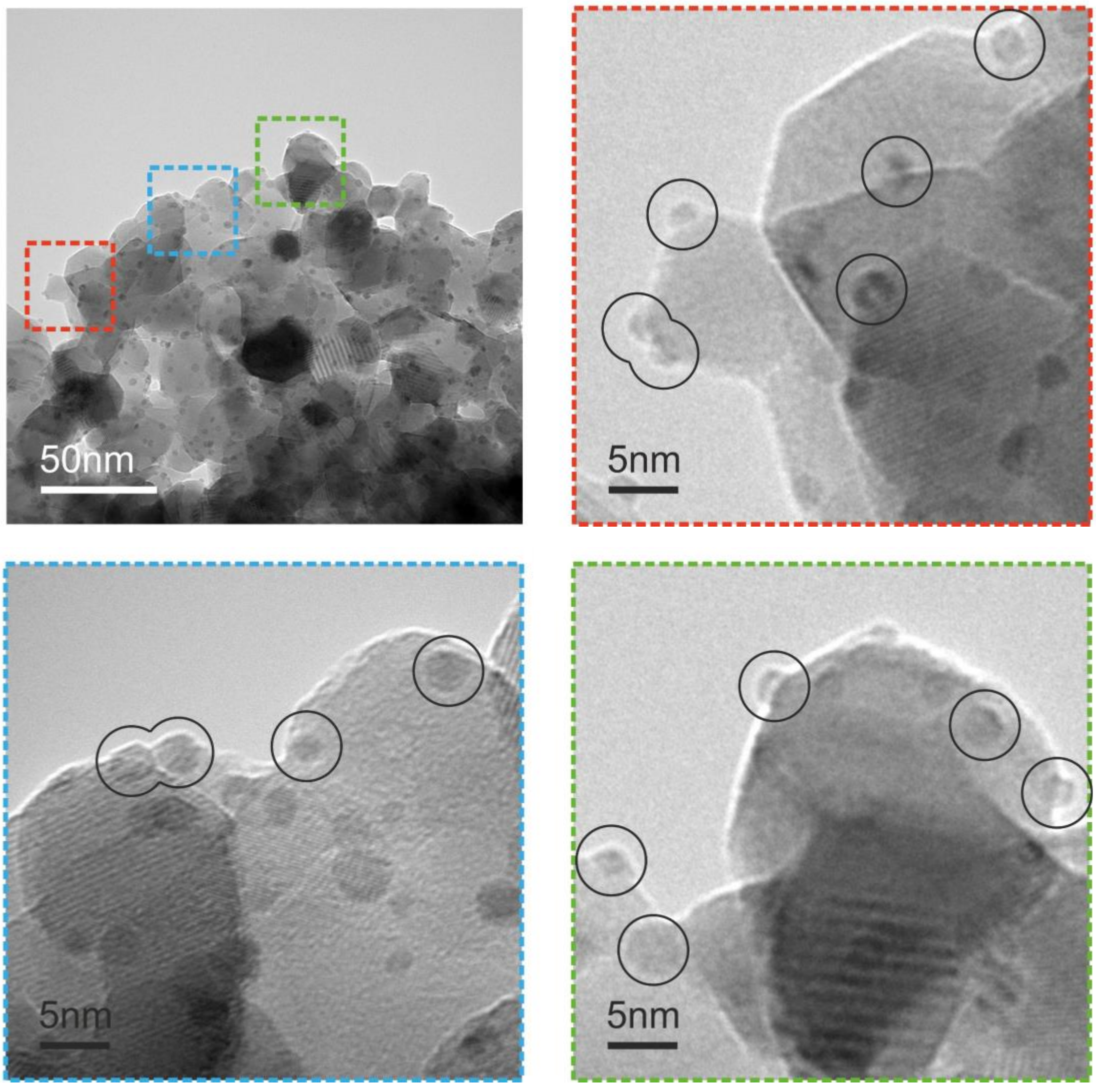

Figure S10. BF-TEM images of reduced Rh-3.0 showing zones of interest with socketed Rh^0 particles at the edges of metal oxide nanoparticles.

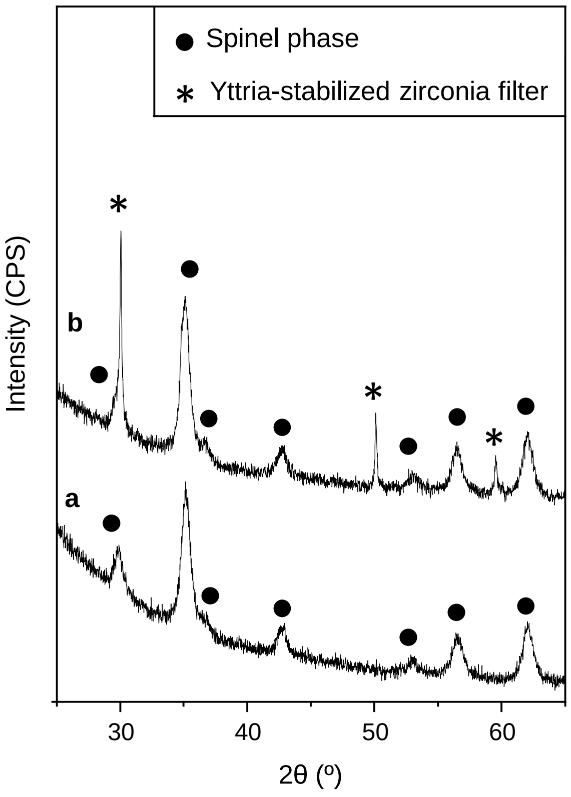

Figure S11. XRD patterns of fresh (a) and reduced (b) Rh-3.0 precursor (reduced at 200 ºC for 2 h in 5% H_2/Ar (30 mL min^{-1}) in the Raman cell).

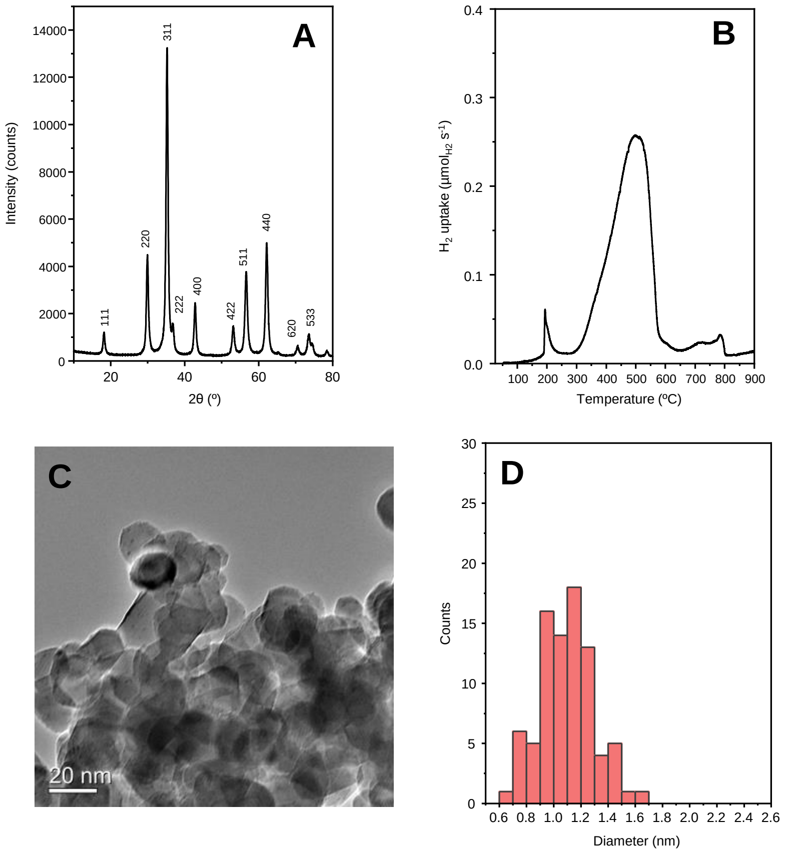

Figure S12. XRD pattern (A) and H_2-TPR profile (B) of the calcined Rh/ZFO sample. HR-TEM image (C) and Rh^0 particle size distribution (D) of Rh/ZFO activated at 200 ºC at 28 bar H_2 for 2 h.

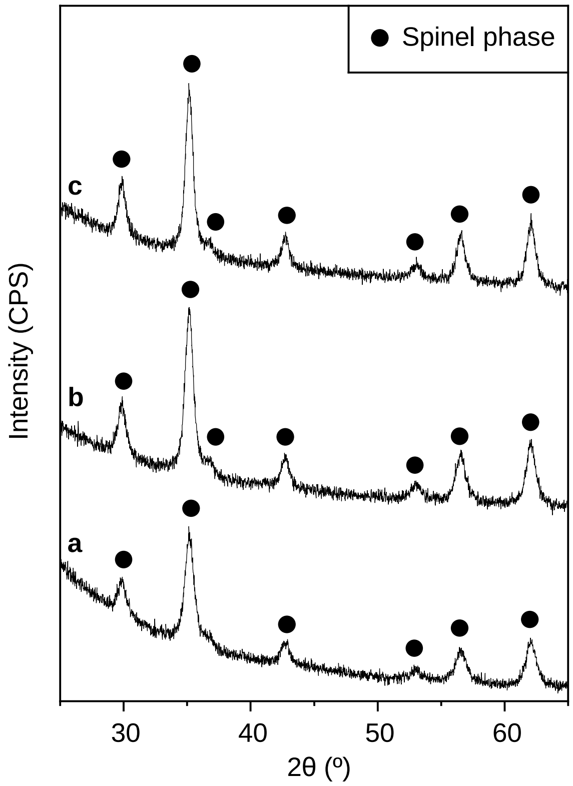

Figure S13. XRD patterns of fresh and spent Rh-3.0 catalysts. a) Calcined Rh-3.0 before hydroformylation of 1-hexene. b) Spent Rh-3.0 catalyst after hydroformylation of 1-hexene at 40 ºC. c) Spent Rh-3.0 sample after the hydroformylation of 1-hexene at 80 ºC.

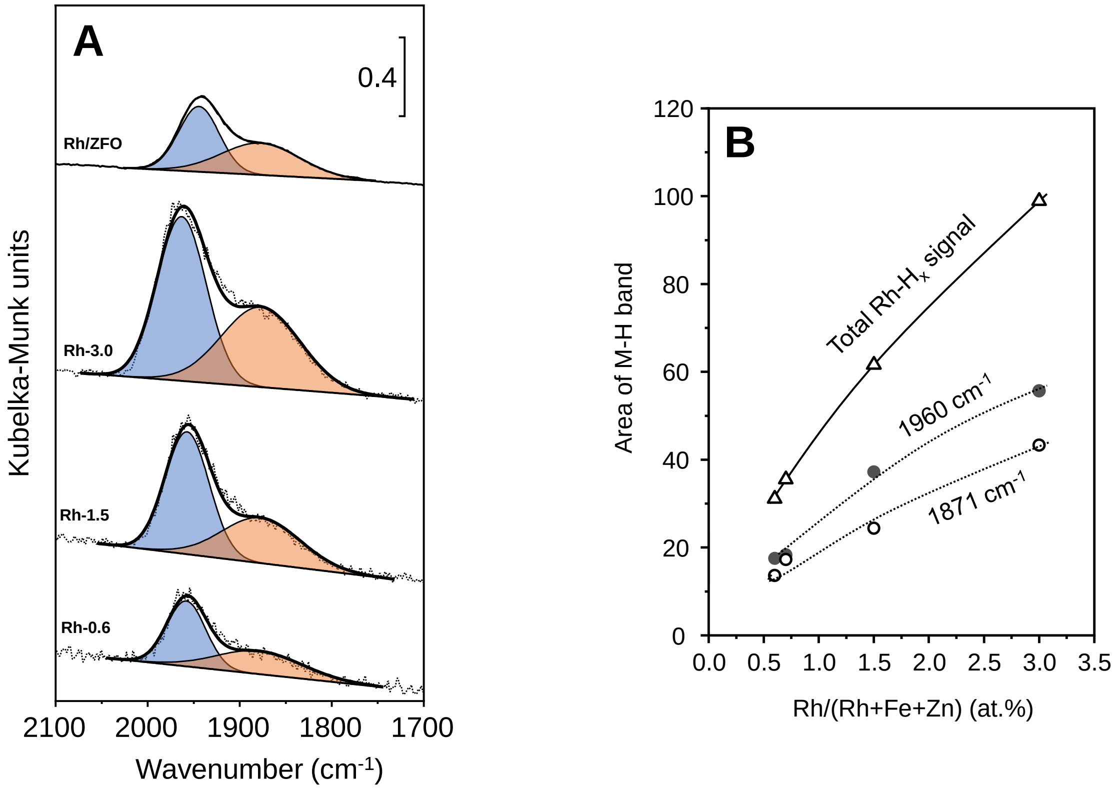

Figure S14. A) Room temperature DRIFT spectra in the R-H_x stretching region (2250-1700 cm^{-1}) of samples Rh-0.6, Rh-1.5, Rh-3.0 and Rh/ZFO activated at 200 ºC in H_2 (28 bar) for 2h. B) Variation of Rh-H_x IR peaks as a function of Rh content in the catalysts.

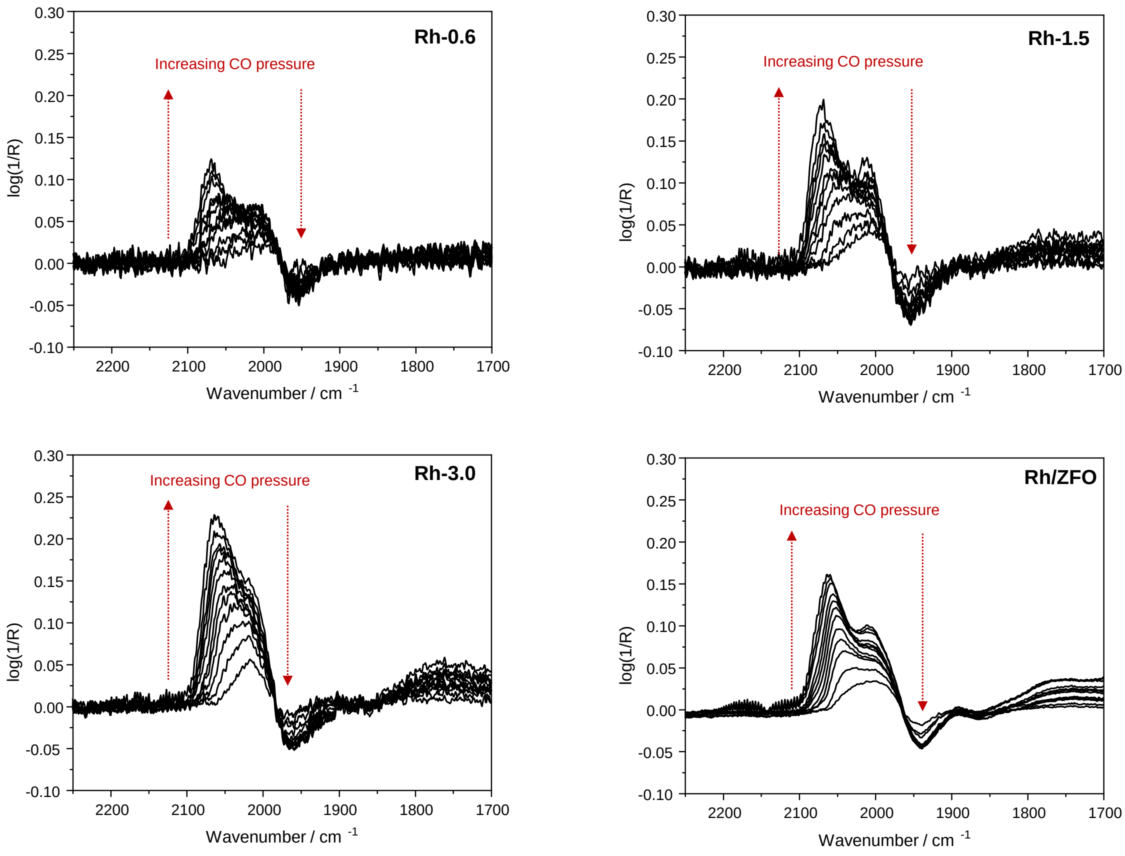

Figure S15. Difference DRIFT spectra of adsorbed CO in the C-O and M-H stretching region for activated Rh-doped catalyst precursors. Activation: 200 ºC in H_2 (28 bar) for 2h.

References

1. Nakagomi, F.; da Silva, S. W.; Garg, V. K.; Oliveira, A. C.; Morais, P. C.; Franco, A., Influence of the Mg-Content on the Cation Distribution in Cubic $Mg_xFe_{3-x}O_4$ Nanoparticles. *Journal of Solid State Chemistry* **2009**, *182*, 2423-2429.

2. Jovanović, S.; Spreitzer, M.; Tramšek, M.; Trontelj, Z.; Suvorov, D., Effect of Oleic Acid Concentration on the Physicochemical Properties of Cobalt Ferrite Nanoparticles. *The Journal of Physical Chemistry C* **2014**, *118*, 13844-13856.

3. Umpierre, A. P.; de Jesús, E.; Dupont, J., Turnover Numbers and Soluble Metal Nanoparticles. ChemCatChem **2011**, *3*, 1413-1418.

4. Galdeano-Ruano, C.; Lopes, C. W.; Motta Meira, D.; Corma, A.; Oña-Burgos, P., Rh_2P Nanoparticles Stabilized by Carbon Patches for Hydroformylation of Olefins. *ACS Applied Nano Materials* **2021**, *4*, 10743-10753.

5. Mukhopadhyay, K.; Mandale, A. B.; Chaudhari, R. V., Encapsulated $HRh(Co)(PPh_3)_3$ in Microporous and Mesoporous Supports: Novel Heterogeneous Catalysts for Hydroformylation. *Chemistry of Materials* **2003**, *15*, 1766-1777.

6. Shang, W.; Qin, B.; Gao, M.; Qin, X.; Chai, Y.; Wu, G.; Guan, N.; Ma, D.; Li, L., Efficient Heterogeneous Hydroformylation over Zeolite-Encaged Isolated Rhodium Ions. *CCS Chemistry* **2022**, *5*, 1526-1539.
